# Supplementary figures and images for: DNMT1 loss leads to hypermethylation of a subset of late replicating domains by DNMT3A
Source: PLoS Genet. 2026 Apr 2;22(4):e1012098. doi: 10.1371/journal.pgen.1012098 (PMC13061326; doi:10.1371/journal.pgen.1012098)

Fig. S1

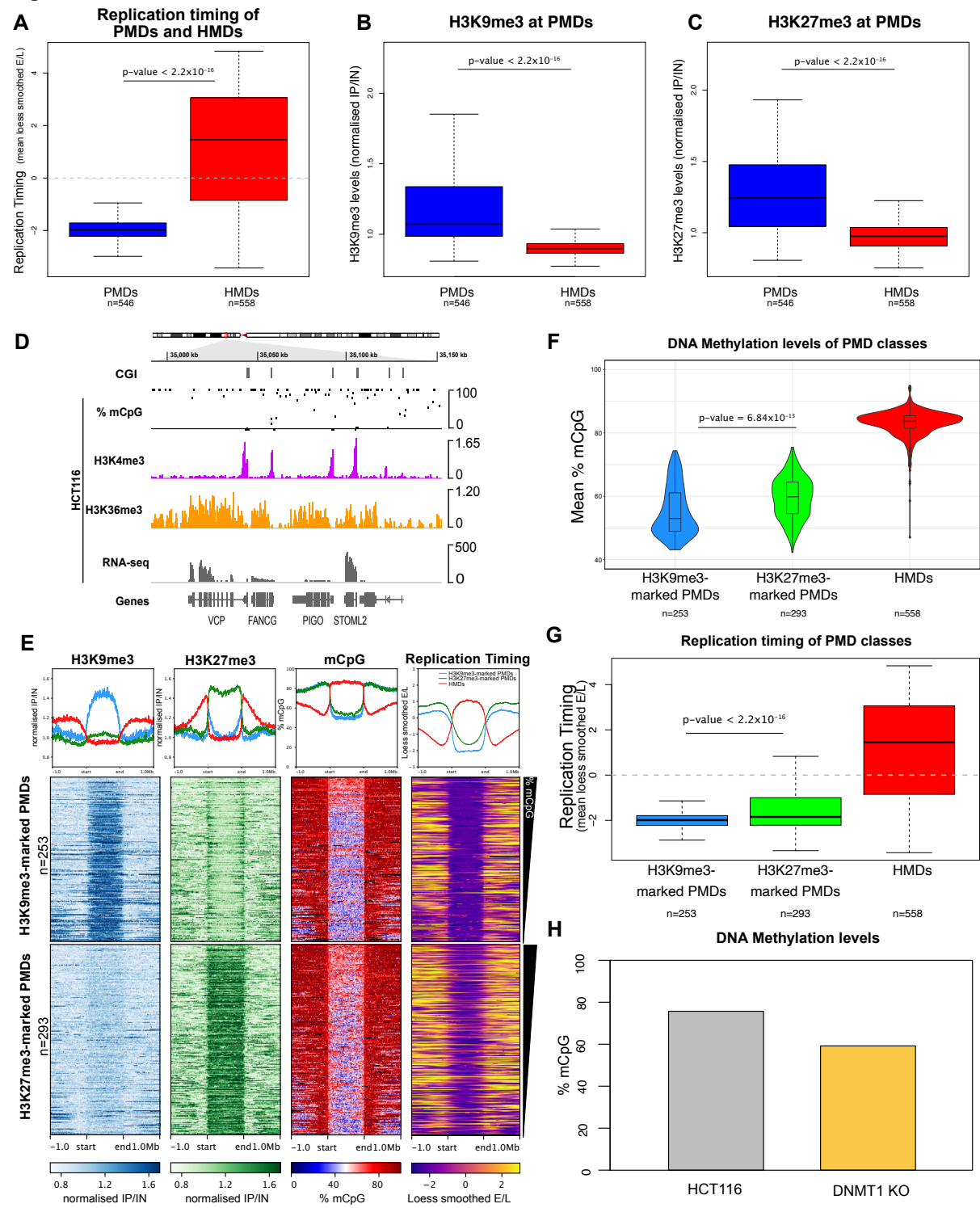

Supplement: S1 Fig — (A) Boxplot showing replication timing of HCT116 PMDs (n = 546 domains) and HMDs (n = 558 domains). Replication timing data are mean loess smoothed repli-seq early/late ratios over 10 kb. (B) Boxplot showing H3K9me3 levels of HCT116 PMDs (n = 546 domains) and HMDs (n = 558 domains). ChIP-seq data are mean normalised IP/IN. (C) Boxplot showing H3K27me3 levels of HCT116 PMDs (n = 546 domains) and HMDs (n = 558 domains). ChIP-seq data are mean normalised IP/IN. (D) Representative genomic locus showing histone marks at genes in HCT116 cells. Genome browser plot showing DNA methylation levels (mC) alongside HCT116 histone modification ChIP-seq and gene expression. DNA methylation levels are plotted for individual CpGs with coverage ≥ 5. ChIP-seq are normalised log10 IP/IN. RNA-seq are mean logRPKM. CGI = CpG islands. Chromosomal co-ordinates shown are hg38 chr9:35,000,000–35,150,000 (E) Heatmaps and pileup plots of HCT116 H3K9me3, H3K27me3 and DNA methylation levels alongside replication timing for H3K9me3-marked PMDs (n = 253 domains) and H3K27me3-marked PMDs (n = 293 domains). ChIP-seq data are mean normalised IP/IN, DNA methylation levels are mean % mCpG over 10kb. Replication timing data are mean loess smoothed repli-seq early/late ratios over 10kb. PMDs are aligned and scaled to the start and end points of each domain and ranked based on their mean methylation levels in HCT116 cells. (F) Violin plot showing mean HCT116 DNA methylation levels at H3K9me3 PMDs (n = 253 domains), H3K27me3 PMDs (n = 293 domains) and HMDs (n = 558 domains). (G) Boxplot showing replication timing of HCT116 H3K9me3 PMDs (n = 253 domains), H3K27me3 PMDs (n = 293 domains) and HMDs (n = 558 domains). Replication timing data are mean loess smoothed repli-seq early/late ratios over 10 kb. (H) Total DNA methylation levels are reduced in DNMT1 KO cells. Barplot of total methylated CpG levels estimated by WGBS. For boxplots: Lines = median; box = 25th–75th percentile; whiskers = 1.5 × interquartile [file pgen.1012098.s001.pdf]

Fig. S4

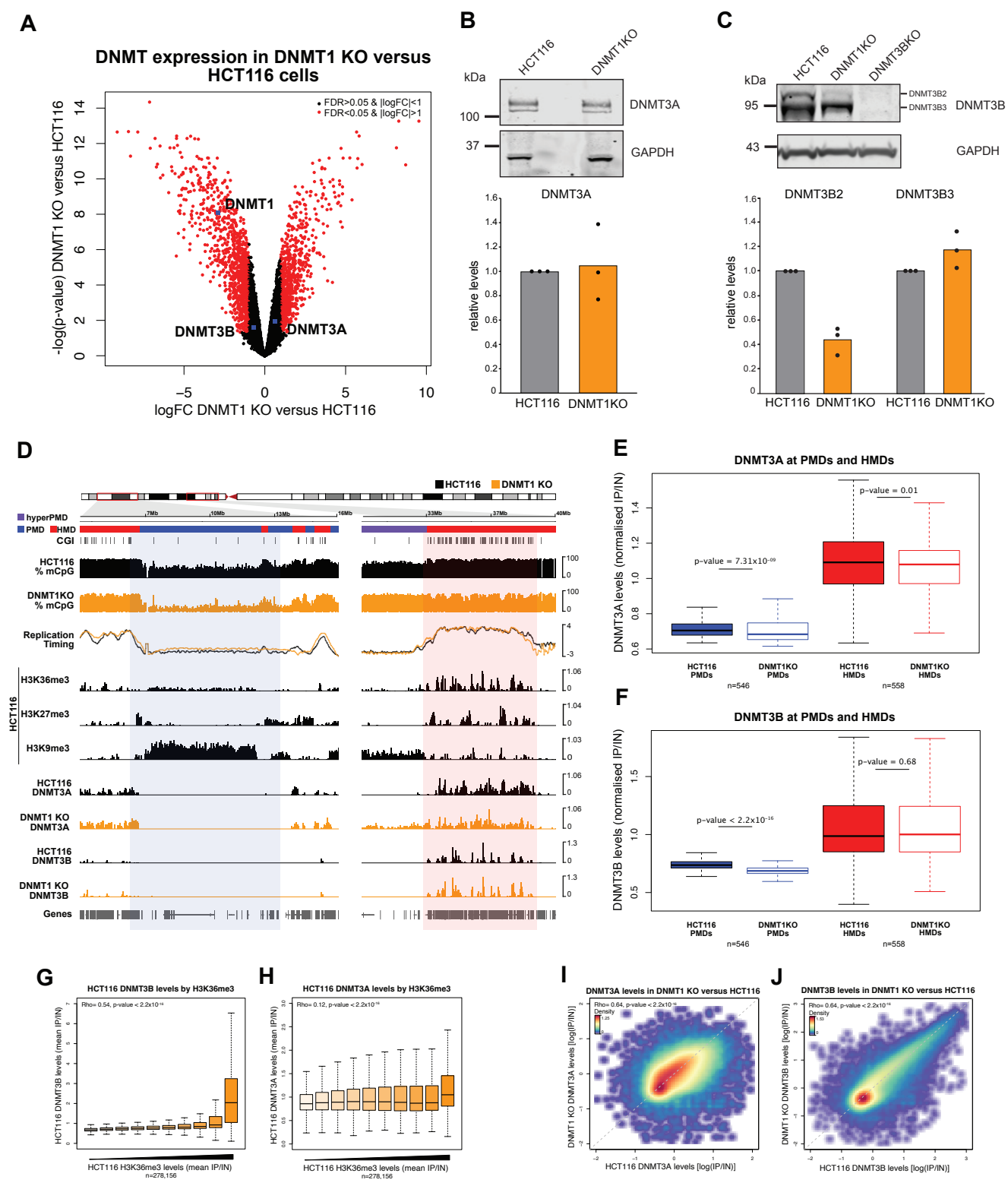

Supplement: S4 Fig — (A) Volcano-plot showing differential expression of protein coding genes between HCT116 and DNMT1KO cells. DNMT1, DNMT3A and DNMT3B are indicated. FC = fold change. (B,C) Western blots of DNMT3A (B) and DNMT3B (C) protein levels in HCT116 and DNMT1 KO cells. In both, Western blots are a representative example. Bar heights indicate mean levels in protein extracts from 3 independent cell cultures normalised to HCT116 cells. Individual points indicate the level of each replicate. DNMT3BKO = DNMT3B knockout HCT116 cells to confirm antibody specificity. Uncropped blots are provided in S10 Fig. (D) Representative genomic loci showing DNMT3A/B localisation at a H3K9me3-marked PMD and an HMD in DNMT1 KO cells. Genome browser plots showing DNA methylation levels (mCpG) alongside DNMT3A/B ChIP-seq and HCT116 histone modifications and repli-seq. DNA methylation levels are plotted in 10 kb genomic windows. ChIP-seq tracks are normalised log10 IP/IN. Replication timing data are loess smoothed repli-seq early/late ratios over 10 kb. Representative H3K9me3-marked PMD and HMD are indicated by the coloured boxes. CGI = CpG islands. Chromosomal co-ordinates shown are from hg38, left: chr9:4,000,000–16,000,000 and right: chr9:31,000,000–40,000,000. (E) Boxplot comparing DNMT3A levels at PMDs (n = 546 domains) and HMDs (n = 558 domains). ChIP-seq data are mean normalised IP/IN. P-value from two-sided Wilcoxon rank sum test. (F) Boxplot comparing DNMT3B levels at PMDs (n = 546 domains) and HMDs (n = 558 domains). ChIP-seq data are mean normalised IP/IN. P-value from two-sided Wilcoxon rank sum test. (G) Boxplot showing mean HCT116 DNMTB levels in 10 kb genomic windows divided in deciles according to their mean levels of H3K36me3 in HCT116 cells. ChIP-seq data are normalised IP/IN. Spearman’s correlation coefficient (Rho) is shown alongside its associated p-value and n is the number of windows analysed. (H) Boxplot showing mean HCT116 DNMT3A levels in 10 kb genomic windows divided in dec [file pgen.1012098.s004.pdf]

**Fig. S5**

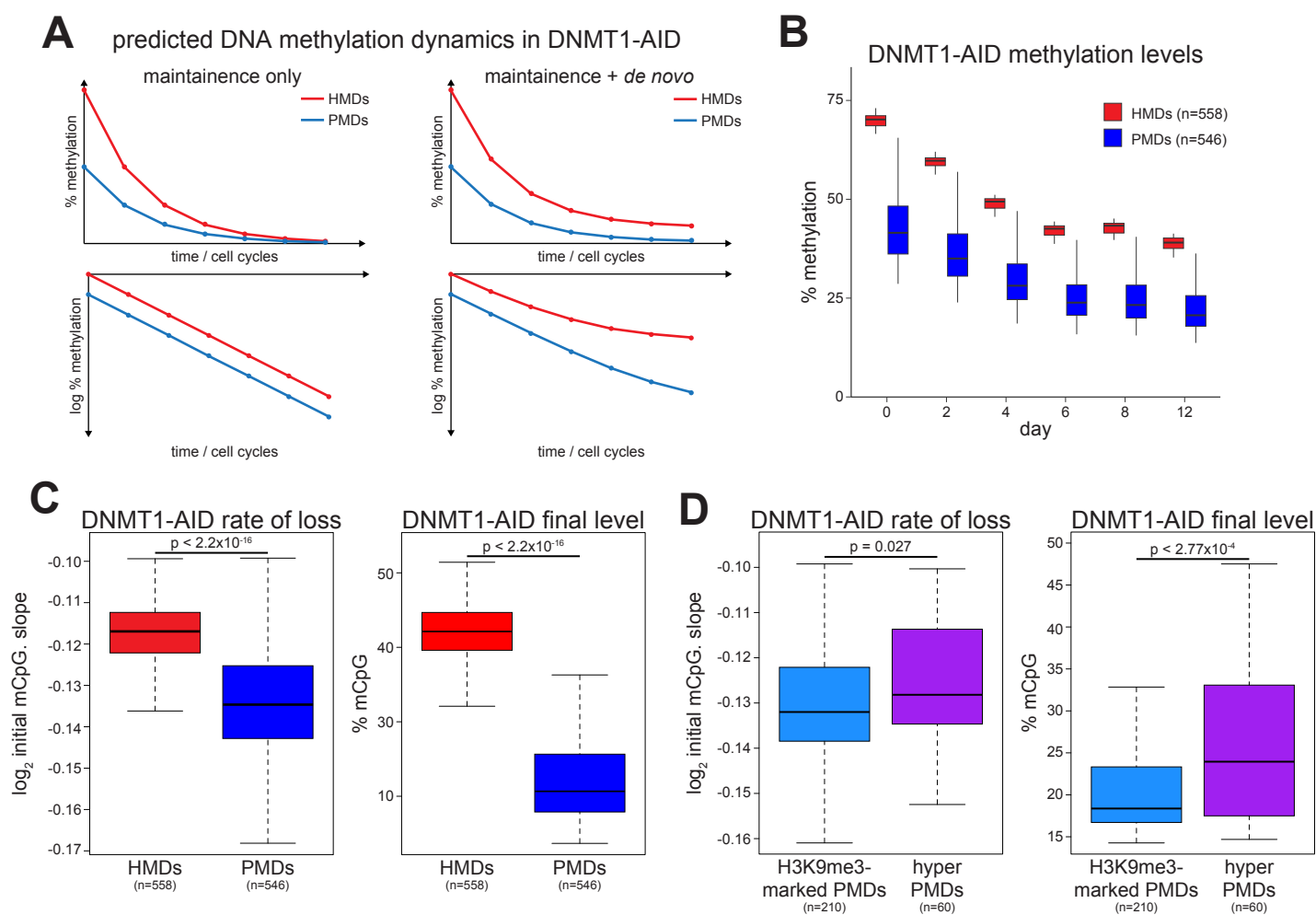

Supplement: S5 Fig — (A) Schematics illustrating predicted trajectories of DNA methylation levels following DNMT1 removal without (left) and with (right) differential de novo methyltransferase activity. Without de novo DNMT activity both will show exponential decay with an equivalent slope in log space (left). De novo methyltransferase activity will alter both the rate of decay and final plateaux level with higher de novo DNMT activity leading to slower loss and a higher final level. (B) Boxplots of mean methylation level for PMDs and HMDs following degradation of DNMT1 in AID-DNMT1 cells. (C) Boxplots of rate of loss of DNA methylation (left) and final levels of DNA methylation (right) in AID-DNMT1 cells for PMDs and HMDs. (D) Boxplots of rate of loss of DNA methylation (left) and final levels of DNA methylation (right) in AID-DNMT1 cells for H3K9me3-marked and hypermethylated PMDs. In plots, n = 558 domains for HMDs, 546 domains for PMDs, 210 domains for H3K9me3-marked PMDs and 60 for hypermethylated PMDs. For boxplots, lines = median; box = 25th–75th percentile; whiskers = 1.5 × interquartile range from box. All p-values are from two-sided Wilcoxon rank sum tests. (PDF) [file pgen.1012098.s005.pdf]

Fig. S6

A

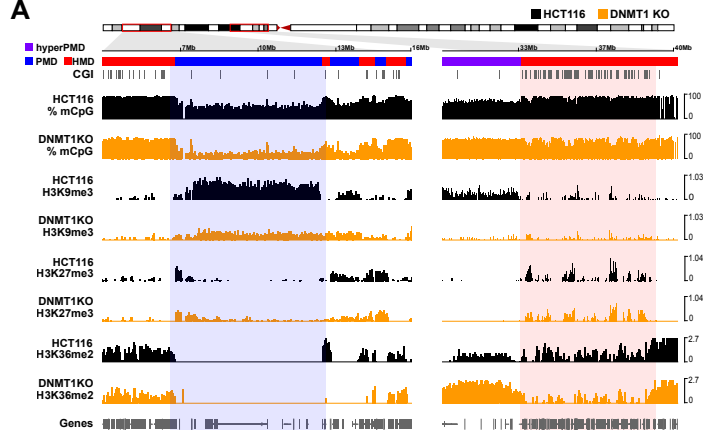

B

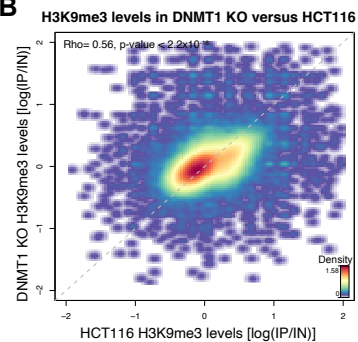

C

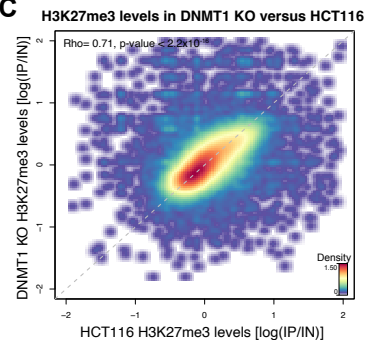

D

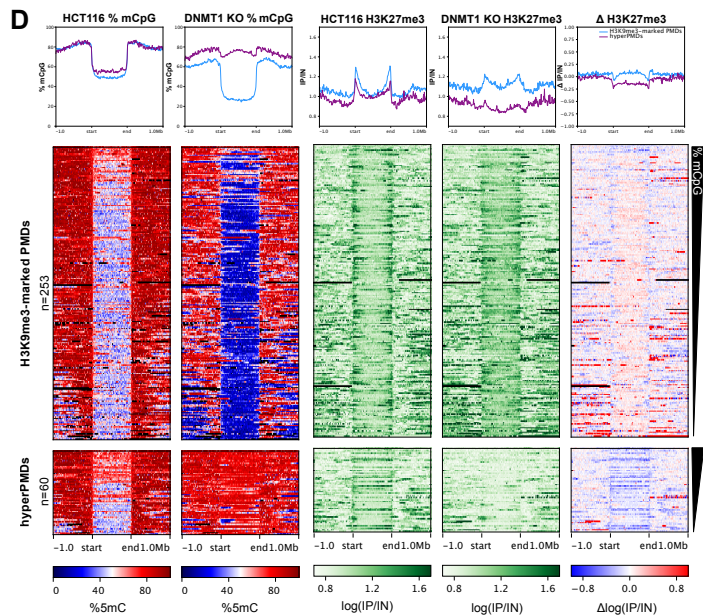

Supplement: S6 Fig — (A) Representative genomic loci showing H3K9me3 and H3K36me2 levels at a representative H3K9me3-marked PMD and an HMD in DNMT1 KO cells. Genome browser plots showing DNA methylation levels (mCpG) alongside H3K9me3, H3K27me3 and H3K36me2 ChIP-seq. DNA methylation levels are plotted in 10 kb genomic windows. ChIP-seq tracks are normalised log10 IP/IN. Representative hypermethylated PMD is indicated by the coloured box. CGI = CpG islands. Chromosomal co-ordinates shown are from hg38: chr9:18,000,000–30,000,000. (B, C) Density scatter plots showing genome-wide correlation H3K9me3 (B) and H3K27me3 (C) levels in DNMT1 KO and HCT116 cells. ChIP-seq data are normalised log IP/IN in 10kb windows. Spearman’s correlations (Rho) and associated p-values are shown. (D) Heatmaps and pileup plots of HCT116 and DNMT1 KO DNA methylation levels alongside H3K27me3 levels for hypermethylated PMDs (n = 60 domains) and all other H3K9me3-marked PMDs (n = 253 domains). ChIP-seq data are mean normalised log IP/IN. DNA methylation levels are mean % mCpG. PMDs are aligned and scaled to the start and end points of each domain and ranked based on their mean methylation levels in HCT116 cells. All histone ChIP-seq data shown are derived from the mean of two biological replicates. (PDF) [file pgen.1012098.s006.pdf]

Fig. S7

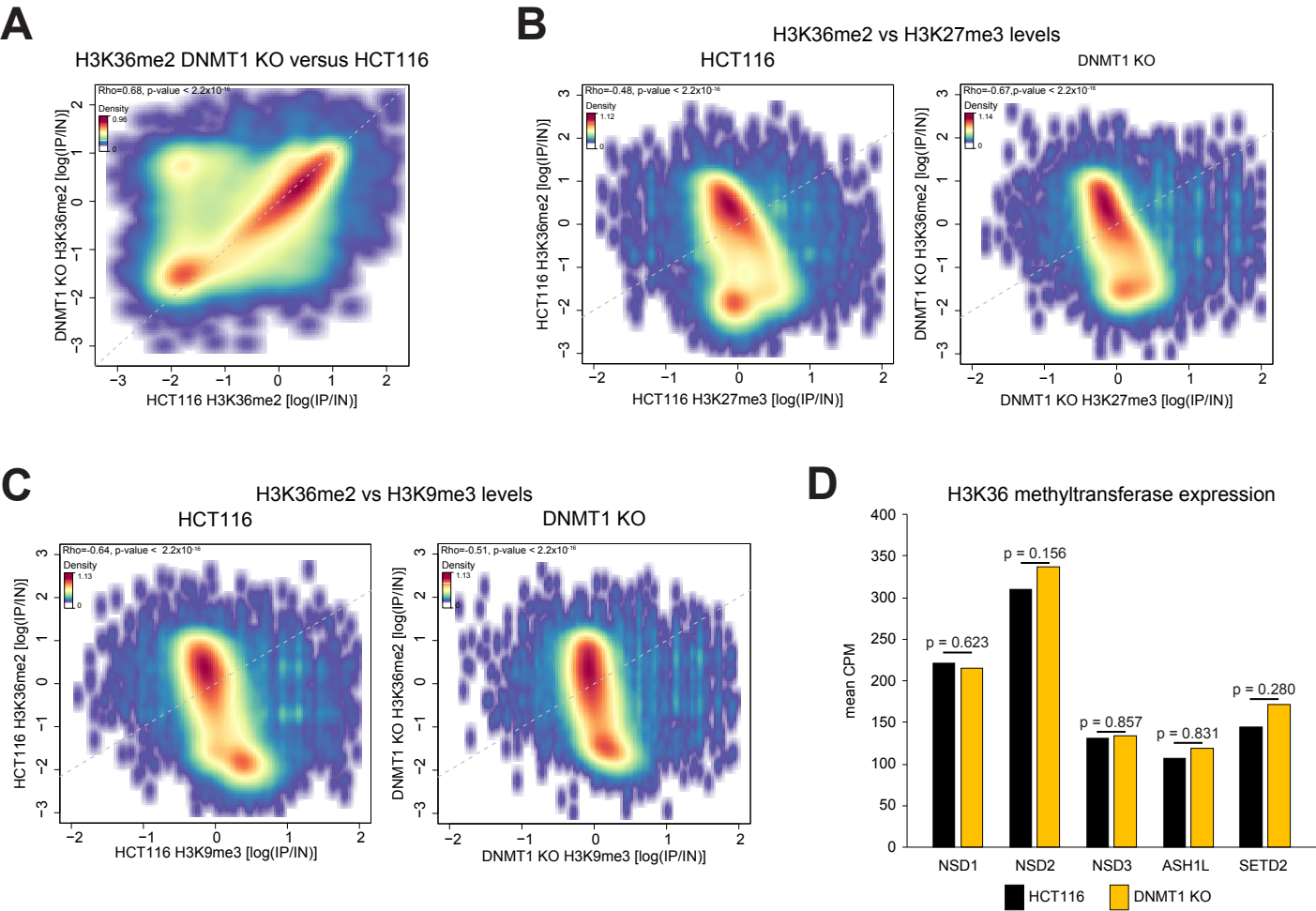

Supplement: S7 Fig — (A) Density scatter plot showing genome-wide correlation H3K36me2 levels in DNMT1 KO and HCT116 cells. (B) Density scatter plots showing genome-wide correlation between H3K27me3 and H3K36me2 levels in HCT116 (left) and DNMT1 KO cells (left). (C) Density scatter plots showing genome-wide correlation between H3K9me3 and H3K36me2 levels in HCT116 (left) and DNMT1 KO cells (left). ChIP-seq data are normalised IP/IN in 10kb windows. In (A-C) Spearman’s correlations (Rho) and associated p-values are shown. All histone ChIP-seq data shown are derived from the mean of two biological replicates. (D) Barplot of RNA-seq derived expression values for known H3K36 methyltransferses in HCT116 and DNMT1 KO cells. Expression values are shown mean Counts Per Million (CPM) calculated form n = 9 and 4 independent cultures of HCT116 and DNMT1 KO cells respectively. P-values shown are Benjami-Hochberg adjusted p-values derived from F-tests. (PDF) [file pgen.1012098.s007.pdf]

**Fig. S8**

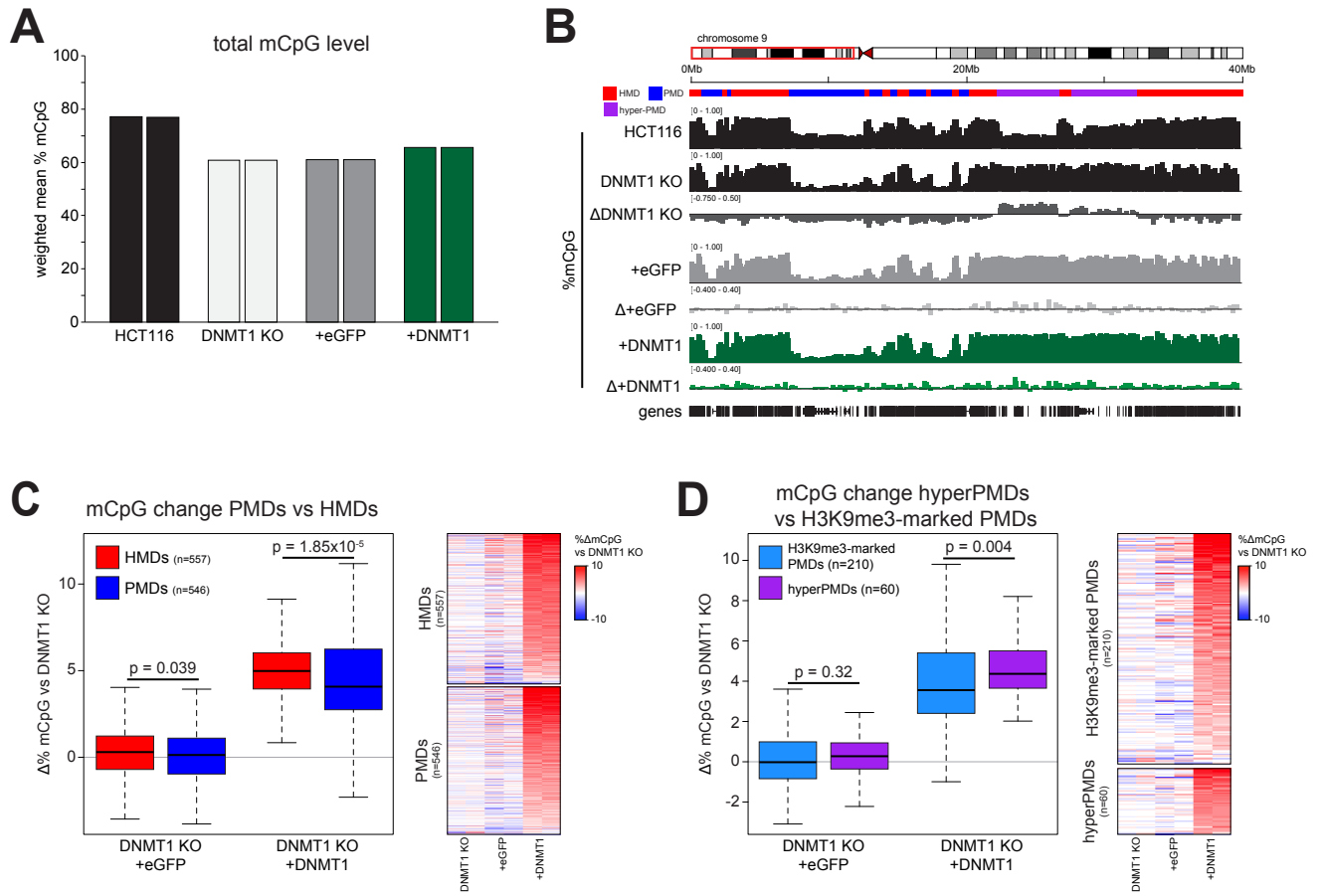

Supplement: S8 Fig — (A) Barplot of total CpG DNA methylation levels estimated by Nanopore sequencing from all aligned reads. For each condition, two independent replicate cultures are shown. (B) Representative genomic locus showing changes of DNA methylation in DNMT1 KO cells expressing ectopic DNMT1. Genome browser plots showing absolute and differential DNA methylation levels. DNA methylation levels are plotted in 10 kb genomic windows and are the mean of n = 2 independent replicate cultures. PMDs and HMDs are indicated by the coloured boxes. Chromosomal co-ordinates shown are hg38 chr9:0–40,000,000. (C, D) Boxplots (left) and heatmaps (right) showing differential DNA methylation between DNMT1 KO and DNMT1 KO cells expressing either eGFP or DNMT1. (C) Comparison of PMDs and HMDs. (D) Comparison of H3K9me3-marked PMDs and hypermethylated PMDs. Boxplots show the mean values of two independent replicate cultures. Heatmaps show replicates separately. In plots, n = 558 domains for HMDs, 546 domains for PMDs, 210 domains for H3K9me3-marked PMDs and 60 for hypermethylated PMDs. In all plots: + eGFP = DNMT1 KO expressing eGFP and +DNMT1 = DNMT1 KO expressing DNMT1. P-values shown are from two-sided Wilcoxon rank sum tests. For boxplots, lines = median; box = 25th–75th percentile; whiskers = 1.5 × interquartile range from box. All p-values are from two-sided Wilcoxon rank sum tests. (PDF) [file pgen.1012098.s008.pdf]

Fig. S9

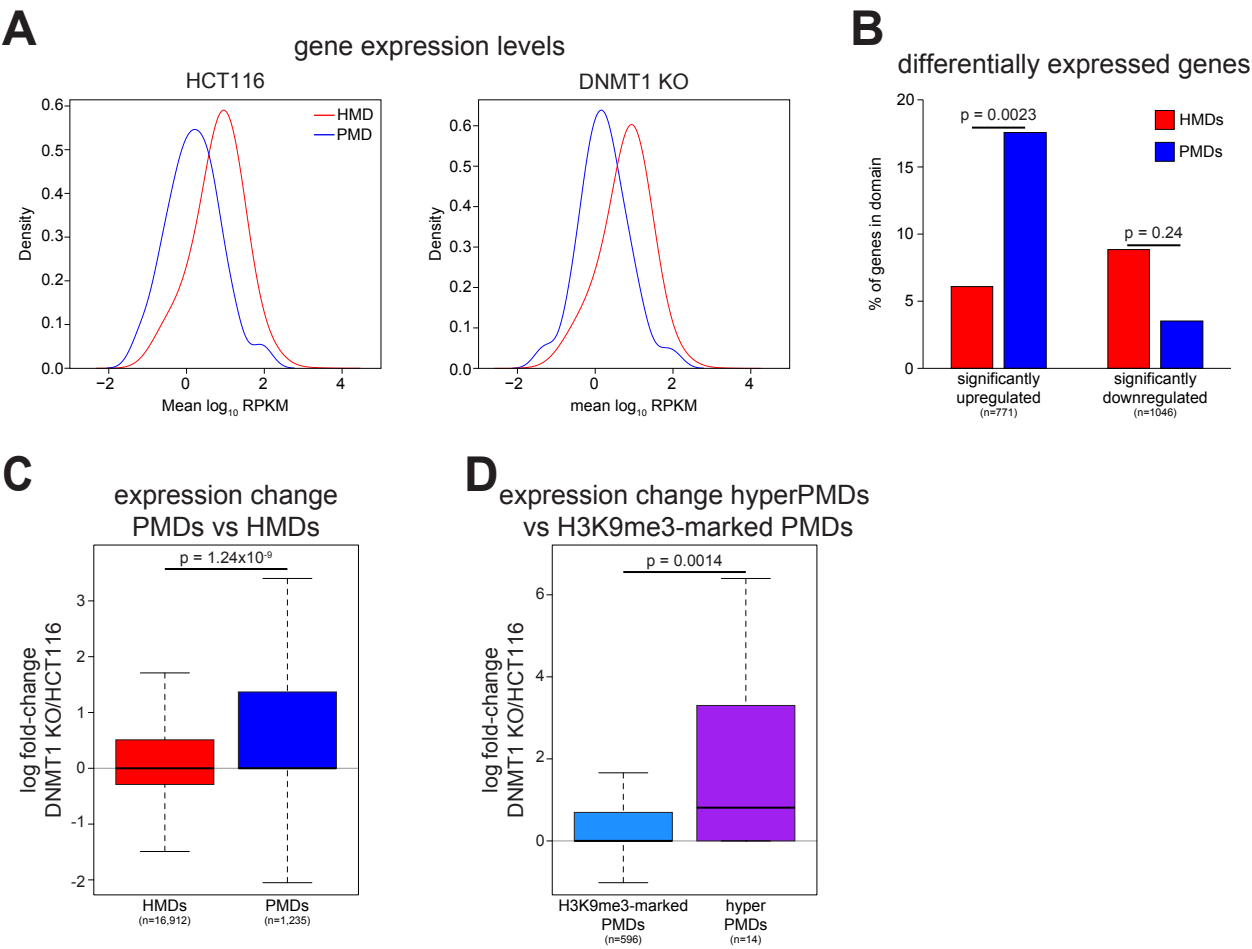

Supplement: S9 Fig — A) Density histograms of log10 RPKM values for PMD and HMD associated genes in HCT116 (left) and DNMT1 KO cells (right). HMDs: n = 11,243 genes and PMDs: n = 57 genes.(B) Barplot showing the % of significantly differentially expressed genes in PMDs and HMDs (n = 771 upregulated and 1,046 downregulated genes). (C, D) Boxplots showing log fold changes for DNMT1 KO vs HCT116 cells for genes located in PMDs versus HMDs (C) and hypermethylated PMDs versus H3K9me3-marked PMDs (D). In all plots, expression values were calculated form n = 9 and 4 independent cultures of HCT116 and DNMT1 KO cells respectively. In (C and D), n = 16,912 genes for HMDs, 1,235 genes for PMDs, 596 genes for H3K9me3-marked PMDs and 14 genes for hypermethylated PMDs. P-values shown are from two-sided Fisher’s exact tests (B) and Wilcoxon rank sum tests (C, D). For boxplots, lines = median; box = 25th–75th percentile; whiskers = 1.5 × interquartile range from box. (PDF) [file pgen.1012098.s009.pdf]

Fig. S10

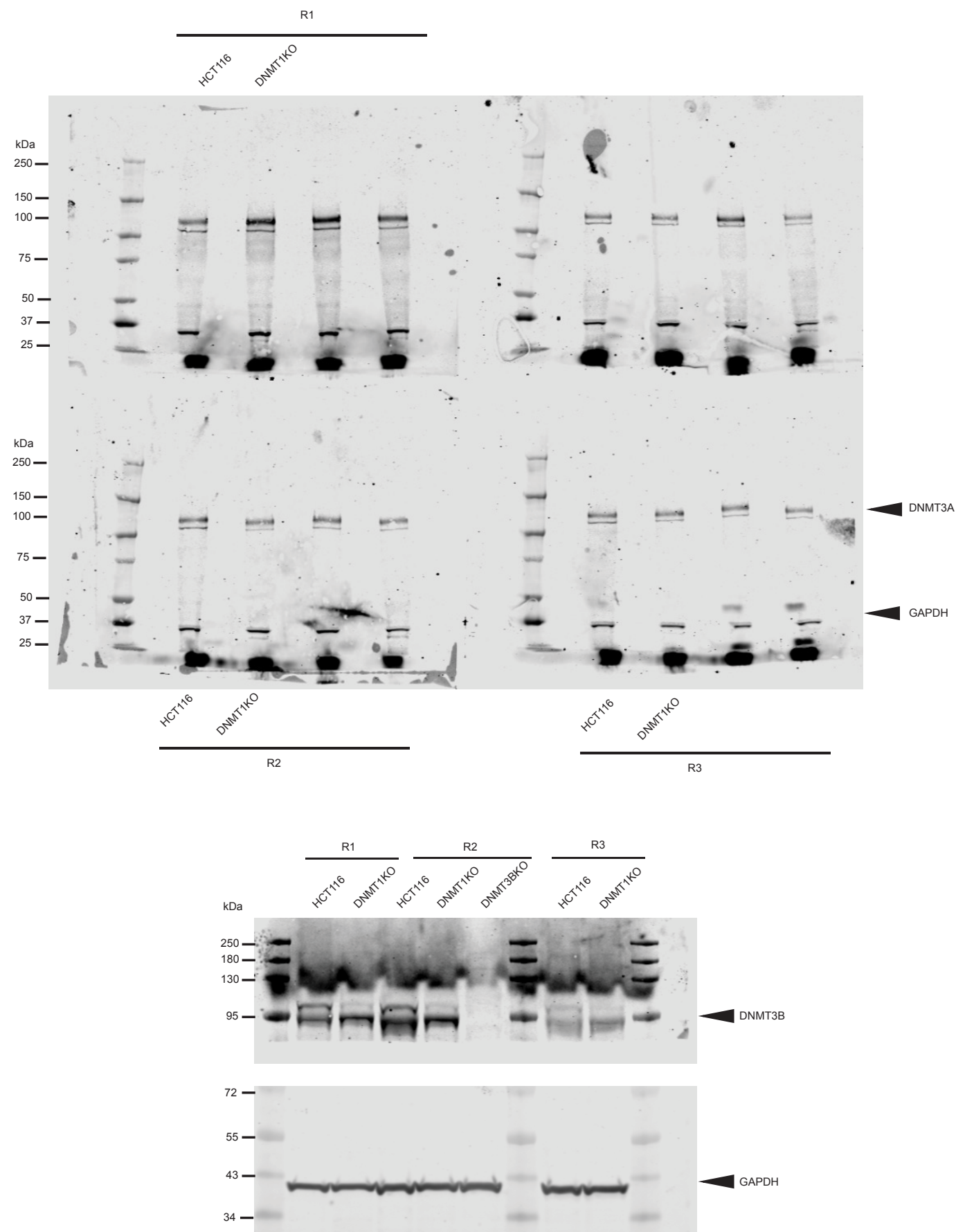

Supplement: S10 Fig — Uncropped scans of the Western blots associated with this study (from S4B-S4C Fig). The corresponding figure number is indicated for each blot. Membranes were cut to probe GAPDH loading controls on the same blot as experimental antibodies. Different exposure times were used for the loading control and experimental antibody. Additional samples not relevant to this study were loaded on some gels and these are not shown in the main figure. (PDF) [file pgen.1012098.s010.pdf]
