# Supplementary material for: DNMT1 loss leads to hypermethylation of a subset of late replicating domains by DNMT3A
Source: PLoS Genet. 2026 Apr 2;22(4):e1012098. doi: 10.1371/journal.pgen.1012098 (PMC13061326; doi:10.1371/journal.pgen.1012098)
Supplement: S2 Fig — (A) Scatter plot of mean methylation levels at PMDs in DNMT1 KO cells versus HCT116 cells highlighting hypermethylated PMDs (hyperPMDs). (B) Ideogram showing genomic distribution and size of hypermethylated PMDs. (C) Boxplot showing HCT116 H3K9me3 levels at hypermethylated PMDs (n = 60 domains) compared to other PMDs (n = 486 domains) and HMDs (n = 558 domains). ChIP-seq data are mean normalised IP/IN. (D) Boxplot showing HCT116 H3K27me3 levels at hypermethylated PMDs (n = 60 Domains compared to other PMDs (n = 486 domains) and HMDs (n = 558 domains). ChIP-seq data are mean normalised IP/IN. (E) Boxplot showing HCT116 replication timing at hypermethylated PMDs (n = 60 domains) compared to other PMDs (n = 486 domains) and HMDs (n = 558 domains). Replication timing data are mean loess smoothed repli-seq early/late ratios over 10kb. For boxplots: Lines = median; box = 25th–75th percentile; whiskers = 1.5 × interquartile range from box. All p-values are from two-sided Wilcoxon rank sum tests. All histone ChIP-seq and repli-seq data shown are derived from the mean of two biological replicates. (PDF) [file pgen.1012098.s002.pdf]

Fig. S2

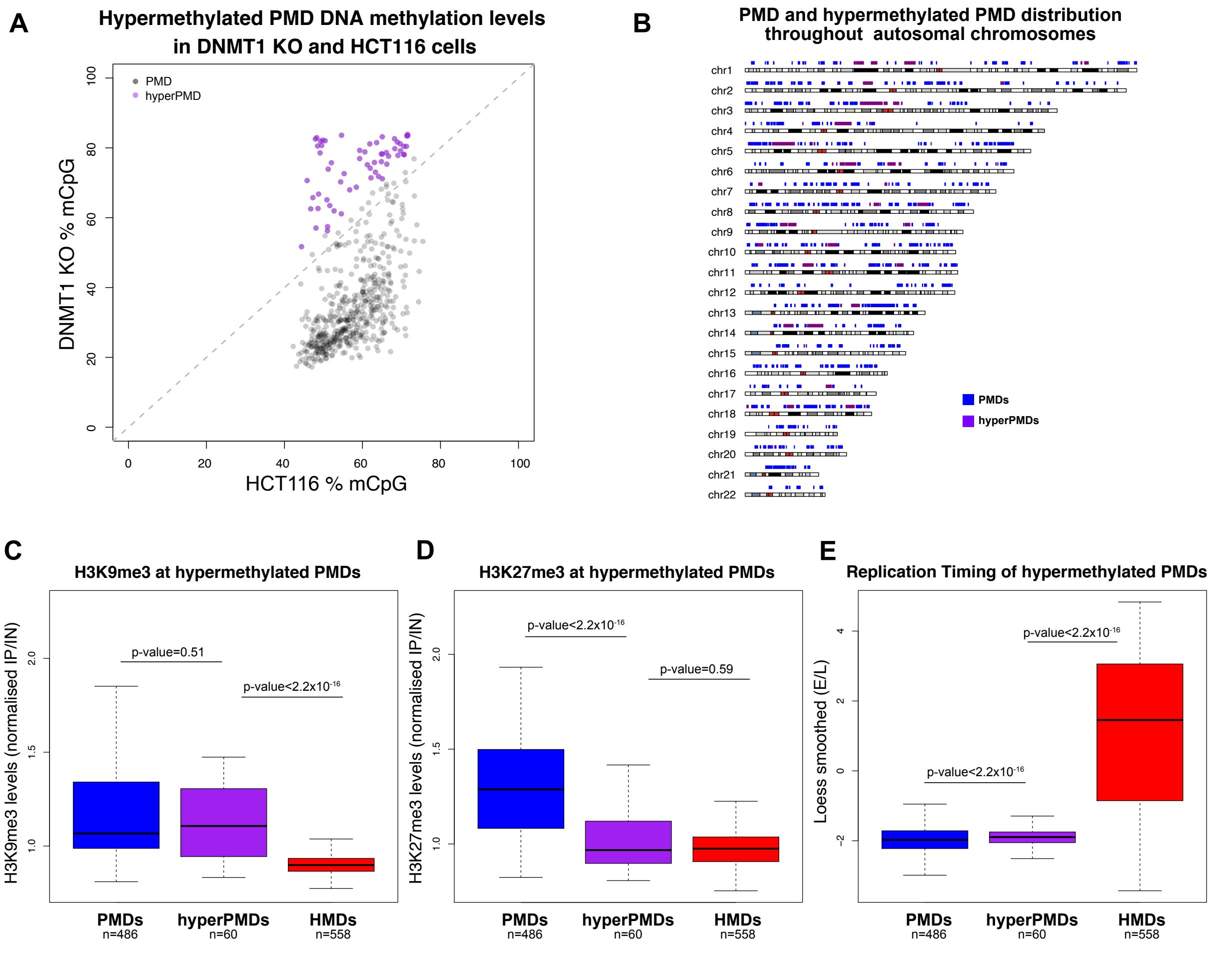
A
Hypermethylated PMD DNA methylation levels in DNMT1 KO and HCT116 cells
PMD
hyperPMD
DNMT1 KO % mCpG
HCT116 % mCpG
0
20
40
60
80
100
B
PMD and hypermethylated PMD distribution throughout autosomal chromosomes
chr1
chr2
chr3
chr4
chr5
chr6
chr7
chr8
chr9
chr10
chr11
chr12
chr13
chr14
chr15
chr16
chr17
chr18
chr19
chr20
chr21
chr22
PMDs
hyperPMDs
C
H3K9me3 at hypermethylated PMDs
H3K9me3 levels (normalised IP/IN)
p-value=0.51
p-value<2.2x10-16
1.0
1.5
2.0
PMDs n=486
hyperPMDs n=60
HMDs n=558
D
H3K27me3 at hypermethylated PMDs
H3K27me3 levels (normalised IP/IN)
p-value<2.2x10-16
p-value=0.59
1.0
1.5
2.0
PMDs n=486
hyperPMDs n=60
HMDs n=558
E
Replication Timing of hypermethylated PMDs
Loess smoothed (E/L)
p-value<2.2x10-16
p-value<2.2x10-16
-2
0
2
4
PMDs n=486
hyperPMDs n=60
HMDs n=558
