# Supplementary material for: DNMT1 loss leads to hypermethylation of a subset of late replicating domains by DNMT3A
Source: PLoS Genet. 2026 Apr 2;22(4):e1012098. doi: 10.1371/journal.pgen.1012098 (PMC13061326; doi:10.1371/journal.pgen.1012098)
Supplement: S3 Fig — (A) Density scatter plot showing genome-wide correlation between replication timing in DNMT1 KO and HCT116 cells. Replication timing data are mean loess smoothed repli-seq early/late ratios in 10kb windows. Spearman’s correlation (Rho) and associated p-value is shown. (B) Boxplot showing HCT116 replication timing in 10 kb genomic windows divided in deciles according to their mean DNA methylation levels in HCT116. Replication timing data are mean loess smoothed repli-seq early/late ratios over 10 kb. Spearman’s correlation coefficient (Rho) is shown alongside its associated p-value and n is the number of windows analysed. (C) Boxplot showing DNMT1 KO replication timing in 10 kb genomic windows divided in deciles according to their mean DNA methylation levels in DNMT1 KO. Replication timing data are mean loess smoothed repli-seq early/late ratios over 10 kb. Spearman’s correlation coefficient (Rho) is shown alongside its associated p-value and n is the number of windows analysed. (D) Boxplot showing replication timing of HCT116 PMDs (n = 486 domains) and HMDs (n = 558 domains). Replication timing data are mean loess smoothed repli-seq early/late ratios over 10 kb. For boxplots: Lines = median; box = 25th–75th percentile; whiskers = 1.5 × interquartile range from box. All p-values are from two-sided Wilcoxon rank sum tests. All repli-seq data shown are derived from the mean of two biological replicates. (PDF) [file pgen.1012098.s003.pdf]

Fig. S3

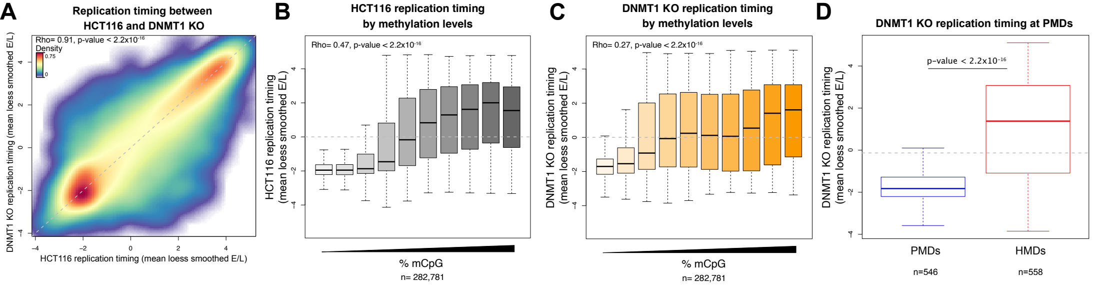
A
Replication timing between
HCT116 and DNMT1 KO
Rho= 0.91, p-value < 2.2x10-16
Density
0.75
0
DNMT1 KO replication timing (mean loess smoothed E/L)
HCT116 replication timing (mean loess smoothed E/L)
B
HCT116 replication timing
by methylation levels
Rho= 0.47, p-value < 2.2x10-16
HCT116 replication timing
(mean loess smoothed E/L)
% mCpG
n= 282,781
C
DNMT1 KO replication timing
by methylation levels
Rho= 0.27, p-value < 2.2x10-16
DNMT1 KO replication timing
(mean loess smoothed E/L)
% mCpG
n= 282,781
D
DNMT1 KO replication timing at PMDs
p-value < 2.2x10-16
DNMT1 KO replication timing
(mean loess smoothed E/L)
PMDs
HMDs
n=546
n=558
