# Supplementary material for: DNMT1 loss leads to hypermethylation of a subset of late replicating domains by DNMT3A
Source: PLoS Genet. 2026 Apr 2;22(4):e1012098. doi: 10.1371/journal.pgen.1012098 (PMC13061326; doi:10.1371/journal.pgen.1012098)
Supplement: Attachment — Submitted filename: 154230_1_rebuttal_3356417_svp89q.pdf [file pgen.1012098.s012.pdf]

Revision Plan

Manuscript number: RC-2025-02860
Corresponding author(s): Duncan, Sproul

[The "revision plan" should delineate the revisions that authors intend to carry out in response to the points raised by the referees. It also provides the authors with the opportunity to explain their view of the paper and of the referee reports.

The document is important for the editors of affiliate journals when they make a first decision on the transferred manuscript. It will also be useful to readers of the reprint and help them to obtain a balanced view of the paper.

If you wish to submit a full revision, please use our "Full Revision" template. ***It is important to use the appropriate template to clearly inform the editors of your intentions.****]*

1. General Statements [optional]

This section is optional. Insert here any general statements you wish to make about the goal of the study or about the reviews.

We thank the reviewers for recognizing that our work contributes 'both conceptually and mechanistically to our understanding of how DNA methylation patterns are regulated during cancer development' and their insightful suggestions to improve the manuscript. We note that the reviewers suggest that the data are 'comprehensive', 'well-controlled', 'rigorously done' and 'diligently analysed'.

Our planned revisions focus on further elucidating the broader implications of our findings for partially methylated domain formation in cancer, the effects of the methylation changes we observe on gene expression and the potential mechanisms underpinning the formation of the hypermethylated domains we observe.

2. Description of the planned revisions

Insert here a point-by-point reply that explains what revisions, additional experimentations and analyses are planned to address the points raised by the referees.

We have reproduced the reviewer's comments in their entirety and highlighted them in *blue italics*.

February 21, 2025 RE: Review Commons Refereed Preprint #RC-2025-02860

Kafetzopoulos

DNMT1 loss leads to hypermethylation of a subset of late replicating domains by DNMT3A

Reviewer #1 (Evidence, reproducibility and clarity (Required)):

The DNA methylation landscape is frequently altered in cancers, which may contribute to genome misregulation and cancer cell behavior. One phenomenon is the emergence of "partially methylated domains (PMDs)": intermediately methylated regions of the genome that are generally heterochromatic and late replicating. The prevailing explanation is that the DNA methyltransferase, DNMT1, is not able to maintain DNAme levels at late replicating sites in proliferating cancer cells. This could result in genome instability. In this study, Kafetzopoulos and colleagues interrogated this possibility using a common laboratory colorectal cancer cell line (HCT116). Additionally, they utilized a DNMT1 mutant line that they refer to as a knockout, even though, more accurately, it is a hypomorphic truncation. They performed several genomic assays, such as whole genome bisulfite sequencing, ChIP and repli-seq, in order to assess the effect of reduced DNMT1 activity. While expectedly, global DNAme levels are decreased, they discovered a subset of PMDs gain DNA methylation, which they term hyperPMDs. There seems to be no impact on DNA replication timing, but the authors did go on to show that the de novo DNA methyltransferase, DNMT3A, is likely responsible for this counterintuitive increase in DNAme levels.

Reviewer #1 (Significance (Required)):

Overall, I found the data well-presented and diligently analyzed, as we have come to expect from the Sproul group. However, I am somewhat at a loss to understand both the rationale for the experimental set-up and the meaning of the results. The HCT116 cell line is already transformed but was treated as though it was a wild-type control. I was more curious to see how the PMD chromatin state and replication compare to a healthy cell.

We focused on the comparison between WT and DNMT1 KO cells as we wanted to understand the role of DNMT1 in maintaining the organisation of the cancer methylome. We agree that, strictly, this could differ from its role in normal cells. However, we are unaware of a suitable cell line to test the consequences of DNMT1 KO in normal colon cells and testing this *in vivo* would be beyond the time-scale of a manuscript revision.

To further understand the relevance of our findings in the context of carcinogenesis, we propose to analyse data derived from normal and cancerous colon tissue in the revised manuscript. Preliminary analysis shows that HCT116 PMDs are hypomethylated in a colorectal tumour but not in the normal colon (*revision plan figure 1*). This suggests that HCT116 cells are a model that can be used to understand PMD formation in tumours and we will extend this analysis in the revised manuscript. We will also add discussion of the caveat that DNMT1 may function differently in normal tissues and cancer cells.

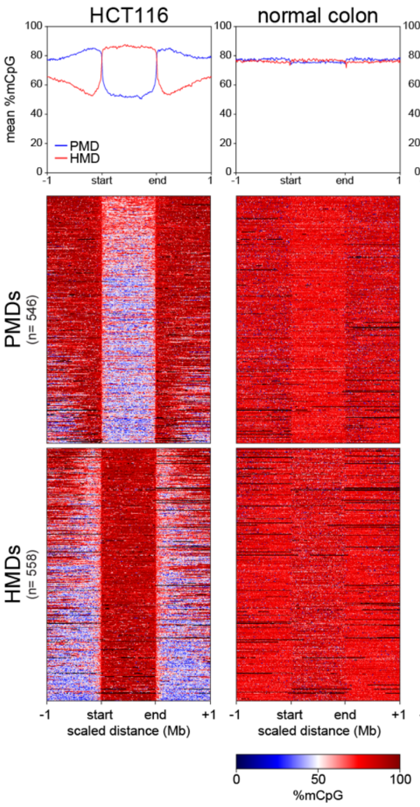

Revision plan figure 1. HCT116 PMDs are hypomethylated in colorectal tumours. Heatmaps and pileup plots of HCT116, normal colon and colorectal tumour DNA methylation levels for HCT116 PMDs (n=546 domains) and HMDs (n=558 domains). DNA methylation levels are mean % mCpG. PMDs and HMDs are aligned and scaled to the start and end points of each domain and ranked based on their mean methylation levels in HCT116 cells. Colon and tumour data re-analysed from a previous publication (Berman et al 2011, PMID: 22120008).

Moreover, the link between late replication and PMDs would indicate that a DNMT1 gain-of- function line would potentially be more interesting: could more increased DNMT activity rescue the PMDs, and how would this impact the chromatin and replication states? Perhaps this is not trivial to create; I do not know if simply overexpressing DNMT1 and/or UHRF1 could act as a gain-of-function.

We agree with the reviewer that a DNMT1 overexpression or a gain-of-function mutation cell line would be interesting to analyse and potentially informative as to the mechanism of PMD formation. However, as the reviewer notes, this is a complex experiment that could require the overexpression of partners such as UHRF1 or generation of an unknown gain-of-function mutation. In addition, the full dissection of the implications of this separate experimental strategy would entail the repetition of the majority of our experiments in DNMT1 KO cells. Instead, in the revised manuscript, we will focus on a related experiment suggested by reviewer 2 and ask whether re-expression of DNMT1 rescues DNA methylation patterns DNMT1 KO cells.

Nevertheless, the appearance of hyperPMDs was a curious finding worth publishing. However, it is unclear what the biological relevance is. There is no effect on replication timing, and no assessment on cell behavior (eg, proliferation assays). In other words, is DNMT3A performing some kind of

compensatory action, or is it just a curiosity? Below in the significance section, I have highlighted some additional specific points

PMDs are important to study because cancer-associated hypomethylation is believed to drive carcinogenesis through genomic instability (Eden et al 2003, PMID: 12702868). However, the mechanisms underpinning their formation remain unclear. At present the predominant hypothesis is that PMDs emerge in heterochromatin because their late replication timing leaves insufficient time for re-methylation following DNA replication (Zhou et al 2018, PMID: 29610480 and Petryk et al 2021, PMID: 33300031). We believe that our observations of hypermethylated PMDs in DNMT1 KO cells provides important evidence contrary to this hypothesis because they disconnect domain-level methylation patterns from the replication timing program. Our work instead suggests that the localization of *de novo* DNMTs plays a key role in the formation of PMDs by protecting euchromatin from hypomethylation.

To further explore this hypothesis, we propose to analyze data derived from tumours in our revised manuscript to understand the degree to which our findings are reflected *in vivo*. As shown above, our preliminary analysis suggests that HCT116 cell PMDs are also hypomethylated in a colorectal tumour but not the normal colon (*revision plan figure 1*). We will also analyze how the changes in methylome affect gene expression using our RNA-seq data.

- Why were DNMT3A and 3B transgenes used for ChIP instead of endogenous proteins? I know the authors cited work justifying this strategy, but this still merits explanation. Also, the expression level of transgenes compared to the endogenes was not shown (neither protein nor RNA level).

DNMT3A and B transgenes were used because antibodies against the endogenous proteins are not suitable for ChIP. Furthermore, performing these experiments using endogenously tagged proteins, required generating 3 knock-in tagged lines (we have already generated HCT116 cells with tagged DNMT3B, Masalmeh et al 2021, PMID: 33514701).

We have previously shown that our constructs do indeed result in overexpression of DNMT3B compared to endogenous protein in this system (Masalmeh et al 2021, PMID: 33514701). However, our previous results also demonstrate that overexpressed DNMT3B recapitulates the localization of the endogenously tagged protein to the genome (Taglini et al 2024, PMID: 38291337). Others have similarly demonstrated that ectopically expressed DNMT3A and DNMT3B can be used to understand their localization on the genome (Baubec et al 2015, PMID: 25607372 and Weinberg et al 2019, PMID: 31485078).

To address this point, we propose to add further justification of our approach and discussion of this potential limitation to a revised version of the manuscript.

- The DNMT3A binding profile appears as though it is on the edges of the PMDs and fairly depleted within (Fig 4A,D). Could the authors comment on this?

This is an interesting point. We note that although mean DNMT3A signal is indeed higher at the edges of hypermethylated PMDs than inside these domains, its levels are both above background and the levels observed in HCT116 cells. As suggested by reviewer 3, this could be consistent with H3K36me2 and DNMT3A spreading in from the boundaries of hypermethylated PMDs in DNMT1 KO cells. We propose to add discussion of this possibility to the revised version of the manuscript.

- A more compelling experiment would be to assess the loss of DNMT3A genetically. How would this affect PMD DNA methylation? Maybe in this case there would be an effect on replication timing. Could a KO or KD (eg, siRNA) strategy be employed to assess this on top of either the HCT116 or DNMT1 KO.

As the reviewer suggests, functional experiments aimed at understanding the role of DNMT3A in our system are likely to be informative. We therefore propose to include such experiments in a revised version of the manuscript.

- What is the major H3K36me2 methylatransferase in these cells? Could an Nsd1 KO or KD strategy be used, for example, to show that indeed H3K36 methylation is required for HyperPMDs? This would complement the DNMT3A experiment above.

H3K36 methylation is thought to be deposited in the mammalian genome by at least 8 different methyltransferase enzymes, NSD1, NSD2, NSD3, ASH1L, SETD2, SETMAR, SMYD2 and SETD3 (Wagner and Carpenter 2023, PMID: 22266761). To understand which of these might be responsible for the deposition of H3K36me2 in hypermethylated PMDs, we have examined their expression in HCT116 and DNMT1 KO cells using our RNA-seq data. This suggests that 5 of these enzymes are highly expressed in HCT116 cells and their expression levels are similar in DNMT1 KO cells: NSD1, NSD2, NSD3, ASH1L and SETD2 (*revision plan figure 2*). The other 3 putative methyltransferases have lower expression levels and, although SMYD2 is significantly upregulated in DNMT1 KO cells, its expression remains low (*revision plan figure 2*). It is currently unclear whether SMYD2 is a bona fide H3K36 methyltransferase (Wagner and Carpenter 2023, PMID: 22266761). We also note that in a recent study, cells lacking NSD1, NSD2, NSD3, ASH1L and SETD2 had no detectable H3K36 methylation, although expression levels of SMYD2 were not reported (Shipman et al, 2024. PMID: 39390582). Based on this analysis, it is therefore unclear which enzyme(s) might be responsible for H3K36me2 deposition in hypermethylated PMDs and delineation of this enzyme would require multiple perturbation and sequencing experiments. We therefore suggest that assessing the consequences of knocking out H3K36me2 methyltransferase activity on hypermethylated PMDs is beyond the scope of a manuscript revision. We propose to include discussion of the expression of the different H3K36me2 depositing enzymes in the revised manuscript.

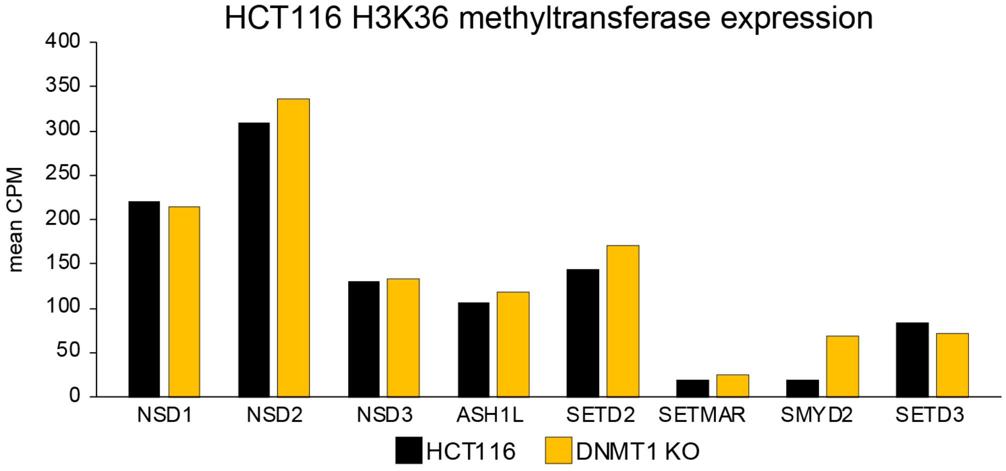

Revision plan figure 2. HCT116 cells express multiple H3K36 methyltransferases. Barplot of mean expression levels for putative mammalian H3K36 methyltransferases in HCT116 and DNMT1 KO cells.

Expression levels are counts per million (CPM) derived from RNA-seq. Mean expression levels are derived from 9 and 4 independent cultures of HCT116 and DNMT1 KO cells respectively.

- Based on Figure 2C, it seems that a general predictive pattern of hyperPMDs is H3K9me3-enriched and H3K27me3-depleted. Is this an accurate interpretation? Given the authors' expertise in the relationship between DNMT3A and polycomb, could they perhaps give an explanation for this phenomenon?

The reviewer is correct. In HCT116 cells, those PMDs that become hypermethylated in DNMT1 KO cells are marked by H3K9me3 and are H3K27me3-depleted (except at their boundaries). DNMT3A is recruited to polycomb-marked regions associated with H3K27me3 through interaction of its N-terminal region with H2AK119ub. However, this mark is depleted from hypermethylated-PMDs in DNMT1 KO cells (current manuscript *Figure S5D*) meaning that this pathway of recruitment is unlikely to explain DNMT3A's localisation to these regions in DNMT1 KO cells. This is discussed in the current manuscript:

We and others have reported that DNMT3A is also recruited to the polycomb-associated H2AK119ub mark through its N-terminal region (Chen et al, 2024; Gretarsson et al, 2024; Gu et al, 2022; Wapenaar et al, 2024; Weinberg et al, 2021). However, we do not observe the polycomb-associated H3K27me3 mark, which is generally tightly correlated with H2AK119ub (Ku et al, 2008), at hypermethylated PMDs suggesting that H2AK119ub does not play a role in the recruitment of DNMT3A to these regions.

Furthermore, DNMT3A's localisation is predominantly driven by its PWWP-dependent H3K36me2 recruitment pathway unless its PWWP domain is mutated (Heyn et al 2019, PMID: 30478443, Sendžikaitė et al 2019, PMID: 31015495, Kibe et al 2021, PMID: 34048432 and Weinberg et al, 2021, PMID: 33986537). Our observations of DNMT3A at hypermethylated PMDs marked by H3K36me2 is therefore consistent with previous findings. We propose to discuss this point in the revised manuscript.

- This is a minor point, but calling the DNMT1 mutant a "KO" seemed a bit misleading, as it is a truncation mutant. Perhaps there is a more accurate way to describe this line.

We propose to amend the manuscript to reflect this point as suggested by the reviewer. To ensure our responses are consistent with the reviewer comments we continue to refer to this line as DNMT1 KO cells in our revision plan.

Reviewer #2 (Evidence, reproducibility and clarity (Required)):

In this study, Kafetzopoulos et al. investigated the role of DNMT1-mediated methylation maintenance in cancer partially methylated domains (PMDs) using DNMT1 knockout HCT116 colorectal cancer cells. They used a range of sequencing-based approaches, including whole-genome bisulfite sequencing (WGBS), chromatin immunoprecipitation sequencing (ChIP-seq), and replication timing sequencing (Repli-seq), to define the dynamics of DNA methylation loss and gain in PMDs during DNA synthesis. Interestingly, they demonstrate that specific PMDs marked by H3K9me3 undergo a gain of DNA methylation in DNMT1-deficient HCT116 cells. This increase in methylation is associated with the loss of H3K9me3, an enrichment of H3K36me2, and the recruitment of DNMT3A. These findings suggest that de novo methyltransferase activity plays a critical role in determining which genomic regions become PMDs in cancer.

The authors use a comprehensive and well-controlled set of sequencing-based techniques. While the sequencing depth for DNA methylation is somewhat limited, the inclusion of multiple biological replicates

strengthens the reliability of the data. The study effectively integrates multiple layers of epigenomic information, providing a nuanced view of PMD regulation in the context of DNMT1 loss.

Overall, this paper provides valuable insights into the epigenetic regulation of PMDs in cancer, and its conclusions are well supported by the data. It significantly advances our understanding of how DNMT1 loss reshapes the epigenome and highlights the interplay between de novo and maintenance methylation mechanisms in cancers.

--

Reviewer #2 (Significance (Required)):

General assessment

-The main strength of the study lies in the clear presentation of the data, which follows a cohesive and well-defined storyline.

-The authors demonstrate that both hypomethylated and hypermethylated domains occur at the late replication stage. They further investigate the dynamics of histone modifications and DNA methylation, focusing on the acquisition and loss of these marks, particularly in relation to DNMT3A and DNMT3B.

Limitation

-Although the study is compelling, its primary limitation is the correlative nature of most of the data. While the high-level representations (e.g., tracks, heat maps) are convincing, the study would have been more informative if it had explored the impact of these changes on a specific set of genes or regions critical to cancer initiation and progression. For example, in the DNMT1 knockout model, how does the loss of H3K9me3, the gain of H3K36me2, and the recruitment of DNMT3A in hypermethylated PMDs affect the expression of key genes involved in colorectal cancer?

To understand how the remodeling of DNA methylation and chromatin structure in DNMT1 KO cells affects gene expression, we propose to include an analysis of our RNA-seq data in the revised manuscript. We will also cross reference these results and our ChIP-seq with lists of colorectal cancer genes.

Additional experiments that could provide deeper insights

-Cross-validation in other cancer cell lines would have enable to define if these signatures are observed beyond HCT116.

As the reviewer suggests, we propose to undertake analyses of additional samples in the revised manuscript to understand how our findings relate to domain-level methylation patterns beyond HCT116 cells. As noted above in response to reviewer 1, our preliminary analysis suggests our findings are relevant for primary colorectal tumours (*revision plan figure 1*).

-Are the observed signatures permanent, or could they be reversed by reinstating the full activity of DNMT1? Since DNMT1 might be dysregulated but never completely deleted.

To address this suggestion, we propose to include the results of a DNMT1 rescue experiment in the revised manuscript.

-Use knockdown and overexpression experiments to track the dynamics and occurrence of these molecular events over time, providing insight into the progression and reversibility of epigenetic changes.

This is an interesting suggestion. As the reviewer suggests, we propose to analyse data derived from time-course experiments to understand the dynamics of changes in different genomic compartments following perturbation of DNMT1.

Advances

-The study provides new insights into the establishment of PMD types in colorectal cancer cell lines.

-These findings contribute both conceptually and mechanistically to our understanding of how DNA methylation patterns are regulated during cancer development.

Audience:

-This study will appeal to a broad audience, from researchers primarily focused on epigenetics and cancer biology to those interested in the mechanistic underpinnings of DNA methylation and its role in cancer progression. It will also be relevant to those exploring therapeutic strategies targeting epigenetic regulators in cancer.

We thank the reviewer for their kind comments on our manuscript.

Reviewer #3 (Evidence, reproducibility and clarity (Required)):

Summary: Cancer is linked to the acquisition of an atypical DNA methylation landscape, with broad domains of partial DNA methylation (termed PMDs). This study investigates PMDs in a colorectal cancer cell line and evaluates the contribution of DNMT1 in maintaining PMDs, using a DNMT1 KO line. The authors find that PMDs preferentially lose DNA methylation upon loss of DNMT1, but they find a number of domains that paradoxically gain DNA methylation (hyperPMDs). They attribute this gain of methylation to the action of DNMT3A through the accumulation of H3K36me2 and loss of heterochromatin mark H3K9me3. Together this work sheds light on the dynamic mechanisms regulating the atypical DNA methylation landscape in colorectal cancer cells.

General comments: The introduction is informative and well written. Additionally, the work is rigorously done and analyses are clear. However, the conclusions and summary figure largely focus on the relationship between PMDs with H3K9me3 and H3K36me2, but I think the role for H3K27me3 should be revisited based on the results presented. H3K9me3 is present at PMDs and hyperPMDs, but H3K27me3 level appears to be a much more defining feature of whether they lose or gain methylation upon loss of DNMT1 (Figure 2, Figure S2C- D). There is a reported interplay between PRC2 and DNMT3A activity at DNA methylation valleys in other cell contexts (e.g., mouse embryogenesis, hematopoietic cells), so couldn't H3K27me3 be performing a 'boundary' function at PMDs and when sufficiently low, permits spread of H3K36me2 in the absence of DNMT1? I think it is worth further exploring the H3K27me3 data.

The reviewer makes an interesting point about the potential for H3K27me3 to act as a boundary preventing H3K36me2 spread into PMDs. Multiple studies have shown that H3K36me2 restricts H3K27me3 deposition in the genome (Streubel et al 2018, PMID: 29606589, Shirane et al 2020, PMID: 32929285 and Farhangdoost et al 2021, PMID: 3362635). The structural nature of this inhibitory effect has also been resolved, demonstrating that the PRC2 catalytic subunit, EZH2 directly binds H3K36 and this is inhibited when the residue is methylated (Jani et al 2019, PMID: 30967505, Finogenova et al 2020, PMID: 33211010 and Cookis et al 2025, PMID: 39774834). The effect of H3K27me3 on H3K36me2 is less well characterised. However, previous work has suggested that inhibiting EZH2 leads to elevated H3K36me2 being established on newly replicated chromatin (Alabert et al 2020, PMID: 31995760). Expression of the EZH2-inhibiting oncohistone H3.3K27M has also been reported to lead to increased H3K36me2 dependent on NSD1/2 in diffuse intrinsic pontine gliomas (DIPG) (Stafford et al 2018, PMID: 30402543 and Yu et al 2021, PMID: 34261657). However, this increase was not reported by an independent study of H3.3K27M DIPG cells (Harutyunyan et al 2020, PMID: 33207202) and the molecular basis of the effect of H3K27me3 on H3K36me2 remains unclear.

As the reviewer suggests, we propose to explore the relationship between H3K27me3 and H3K36me2 further in a revised manuscript along with the including further discussion of previous findings in this area.

Additionally, a key point that is illustrated in the summary figure, is the localization of H3K36me2 at HMDs and its mutual exclusivity with H3K9me3 (a mark typically associated with high DNA methylation). However, because the H3K36me2 is introduced quite late in the analysis, I feel that a rigorous evaluation of its enrichment and anti-correlation with H3K9me3 at highly methylated domains (HMDs) is missing.

The relationship between H3K36me2 and H3K9me3 is far less explored than that of H3K27me3 and H3K36me2. Interestingly, we note that a recent study reported that depletion of H3K36me2 results in H3K9me3 re-distribution suggesting that H3K9me3 is restricted by H3K36me2 (Padilla et al 2024, DOI: 10.1101/2024.08.10.607446, also cited in the original manuscript).

To understand this relationship further, we therefore propose to explore the relationship between H3K9me3 and H3K36me2 in our datasets as part of revised manuscript along with including additional discussion of relevant experimental findings.

In general, I also found that I was jumping between figures a lot and needed to look at the supplements to gain the full picture. It may be beneficial to re-organize the figures.

In accordance with the reviewer's suggestion, we propose to re-organise the revised manuscript to make it easier to follow.

Specific Comments/Questions:

1. An expanded explanation of the truncated DNMT1 in the DNMT1 KO cells would be helpful for context

As suggested by the reviewer, we will amend the manuscript to include an expanded discussion of the DNMT1 truncation present in the cell line.

2. Does the DNMT expression in HCT116 cells reflect the levels seen in primary colorectal cancers? Hence, do you think these cultured cells reflect aspects of DNA methylation dynamics that would be seen in tumors?

While differences between cancer cell line and tumour methylation patterns have previously been noted (for example Anne Rogers et al 2018, PMID: 30559935), we have previously demonstrated that HCT116 cells recapitulate CpG island methylation patterns observed in colorectal tumours (Masalmeh et al 2021, PMID: 33514701). As stated above in response to reviewer 1, we have now examined the methylation status of HCT116 PMDs in a colorectal tumour. This analysis shows that HCT116 PMDs have reduced methylation levels in a colorectal tumour but not in the normal colon (*revision plan figure 1*). We propose to extend this analysis of colorectal tumour samples and add them to the revised manuscript to address this point.

Regarding the expression of DNMTs in colorectal tumours, DNMT1 is ubiquitously expressed to our knowledge. DNMT3B is reported to be overexpressed in 15-20% of cases of colorectal cancer, often as a result of amplification (Nosho et al 2009, PMID: 19470733, Ibrahim et al 2011, PMID: PMID: 21068132, Zhang et al 2018, PMID: 30468428 and Mackenzie et al 2020, PMID: 32058953). DNMT3A expression in colorectal tumours is less studied but one report suggests upregulation in at least some tumours (Robertson et al 1999, PMID: 10325416 and Zhang et al 2018, PMID: 30468428). We propose to add additional discussion of DNMT expression in colorectal cancer to the revised manuscript to clarify the degree to which our results reflect methylation regulation in primary colorectal tumours.

3. Although DNMT3A/B mRNA levels are similar between DNMT1 KO and HCT116 cells, is the protein abundance altered? I think there would be value in showing a Western blot analysis, as the loss of DNMT1 protein may alter the stability of the de novo DNMTs. Is a similar level of expression of the ectopic T7-DNMT3A and T7-DNMT3B achieved in HCT116 and DNMT1 KO cells? A western blot showing this would also be valuable.

As part of our work towards revising the manuscript, we have undertaken blots of DNMT3A in our cell lines. This shows that DNMT3A levels in DNMT1 KO cells are similar to those in HCT116 cells which (*revision plan figure 3*). We propose to include this in the revised manuscript alongside a similar analysis of DNMT3B. We will also include an analysis of T7-DNMT3A and T7-DNMT3B levels to understand whether they are expressed to similar levels in HCT116 and DNMT1 KO cells.

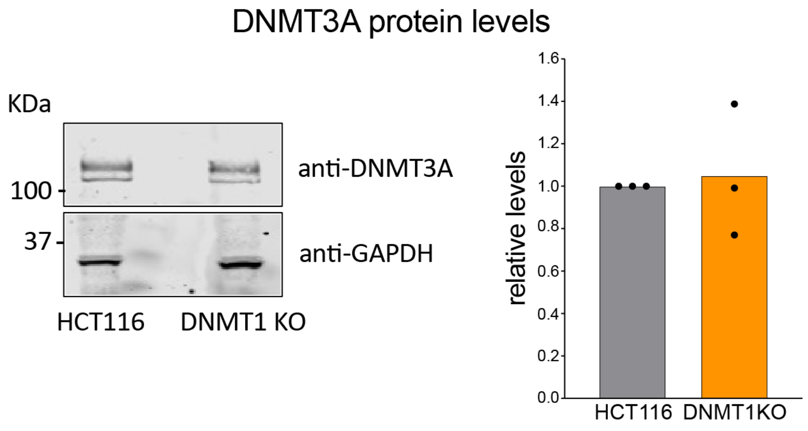

Revision plan figure 3. DNMT3A protein levels are similar in HCT116 and DNMT1 KO cells. Left, representative DNMT3A Western blot. Right, bar plot quantifying relative DNMT3A levels. The bar height indicates the mean levels observed in protein extracts from 3 independent cell cultures. Individual points indicate the level of each replicate.

4. Do you think that the increase in DNMT3A over HyperPMD compared to H3K9me3-marked PMDs is related to an increase in protein bound at these domains or an altered residence time?

The reviewer makes an interesting point with regard to a potential alteration of DNMT3A residence at hypermethylated PMDs. Given that ChIP-seq signal is affected by residence time (Schmiedeberg et al 2009, PMID: 19247482), it is possible that our findings could reflect this rather than increased DNMT3A localisation. We propose to add discussion of this point as a limitation of the current study to the manuscript.

It would also be valuable to move the plot showing levels of DNMT3A/3B at HMDs, from the S4C/D to the main Figure 4, for reference. It would also be interesting to see the enrichment of DNMT3A/B at all PMDs (not just H3K9me3-marked PMDs).

As the reviewer suggests, we will include the data on HMDs to the main *Figure 4* and include enrichments at all PMDs in the supplementary figures.

5. It appears that the same genomic locus is used multiple times across figures Fig 1A, Fig 2B, Fig 3A, Fig 4A, Fig 5B to illustrate the trends reported from the global analyses. While this has value in showing the dynamics across datasets at this region, I think it is important to illustrate that these trends can be observed elsewhere. Please add or replace some plots with additional loci. Furthermore, please add the genomic region coordinates to the figure or figure legend.

We had shown a single locus for consistency and to not overcomplicate figures which already contain multiple panels. As the reviewer suggests, we will add additional loci in the supplementary figures of our revised manuscript. We had also included the chromosome co-ordinates in the figures. In the revised version we will ensure that the precise co-ordinates are included in the legends.

6. The ChIP-seq data is quantified as IP/input. This quantitation can be prone to introducing artefacts into analyses if the input coverage is substantially uneven over AT-rich regions or CpG islands, or if the sequencing depth is insufficient. I would encourage the authors to check that the trends observed are still present if quantified without correcting against the inputs. If using IP/input, in the supplementary figures, I think it would be valuable to show the uncorrected quantitation of inputs across PMDs, to demonstrate that there is even coverage and this isn't contributing to any of the changes observed.

We thank the reviewer for this point and we propose to examine the quantification of the ChIP-seq without normalizing to input to ensure that uneven input signal does not substantially contribute to our results.

7. Generally, the n numbers for different groups of probes can be confusing and increased clarity would be helpful.

We will clarify the explanation of n numbers in the revised manuscript.

Reviewer #3 (Significance (Required)):

This study adds to the accumulating body of evidence that DNMT3A recruitment is mediated primarily through H3K36me2 across cell contexts, shedding light on the interplay between histone modifications and de novo DNA methylation. Understanding these mechanisms is important to appreciate the role for DNMT3A in establishing DNA methylation in development and disease contexts. It does remain unclear

why, upon loss of DNMT1 in colorectal cancer cells, some PMDs accumulate H3K36me2 and consequently DNA methylation, while others do not. Further study into the chromatin dynamics will be valuable in understanding determinants of the DNA methylation landscape in cancer.

We thank the reviewer for their insightful comments and believe that our proposed revisions will further clarify the points they raise.

3. Description of the revisions that have already been incorporated in the transferred manuscript

Please insert a point-by-point reply describing the revisions that were already carried out and included in the transferred manuscript. If no revisions have been carried out yet, please leave this section empty.

We have not yet incorporated revisions into the manuscript.

4. Description of analyses that authors prefer not to carry out

Please include a point-by-point response explaining why some of the requested data or additional analyses might not be necessary or cannot be provided within the scope of a revision. This can be due to time or resource limitations or in case of disagreement about the necessity of such additional data given the scope of the study. Please leave empty if not applicable.

As stated in our responses to the reviewer comments above, we plan to address all comments. However, we suggest that two experiments proposed by the reviewers are beyond the scope of a manuscript revision and we will instead address these comments in the following manner:

A. Analysis of a DNMT1 gain-of-function line (Reviewer 1). As suggested by the reviewer such a line is non-trivial to generate. It would also require extensive profiling of this new line to fully understand its implications for our findings. We therefore believe it is outwith the scope of a manuscript revision. Instead, we propose to address this comment by undertaking the related experiment suggested by Reviewer 2 and perform a DNMT1 rescue experiment in the DNMT1 KO line.

B. Analysis of H3K36me2 methyltransferase knockout cells (Reviewer 1). Our preliminary analysis suggests that HCT116 cells express multiple H3K36 methyltransferases and that their expression does not vary greatly in DNMT1 KO cels (*revision plan figure 2*). This means that it is unclear which enzyme(s) might be responsible for depositing H3K36me2 in hypermethylated PMDs. Delineation of this would require the generation and analysis of multiple knockouts and we suggest it is therefore outwith the scope of a manuscript revision. To address this point we will instead include discussion of the spectrum of H3K36 methyltransferases expressed in our cells in the revised manuscript as detailed in the specific response above.
