# Supplementary material for: DNMT1 loss leads to hypermethylation of a subset of late replicating domains by DNMT3A
Source: PLoS Genet. 2026 Apr 2;22(4):e1012098. doi: 10.1371/journal.pgen.1012098 (PMC13061326; doi:10.1371/journal.pgen.1012098)
Supplement: Attachment — Submitted filename: kafetzopoulos_et_al_rebuttal_20260130.pdf [file pgen.1012098.s013.pdf]

Response to Reviewers

We thank the reviewers for their supportive and insightful comments that have helped us substantially improve the manuscript. In particular, in the revised manuscript we provide additional work showing the relevance of our findings for clinical colorectal tumours, impact on expression and additional controls to reinforce our findings.

We provide a point-by-point response to each comment below. Reviewers' comments are indicated by *blue italic* text. Any positions referenced correspond to those in the revised document. We also enclose a revised manuscript, with changes highlighted in blue.

We have also re-numbered reviewer comments to facilitate cross-referencing between related points.

Reviewer #1 *(Evidence, reproducibility and clarity (Required)):*

The DNA methylation landscape is frequently altered in cancers, which may contribute to genome misregulation and cancer cell behavior. One phenomenon is the emergence of "partially methylated domains (PMDs)": intermediately methylated regions of the genome that are generally heterochromatic and late replicating. The prevailing explanation is that the DNA methyltransferase, DNMT1, is not able to maintain DNAme levels at late replicating sites in proliferating cancer cells. This could result in genome instability. In this study, Kafetzopoulos and colleagues interrogated this possibility using a common laboratory colorectal cancer cell line (HCT116). Additionally, they utilized a DNMT1 mutant line that they refer to as a knockout, even though, more accurately, it is a hypomorphic truncation. They performed several genomic assays, such as whole genome bisulfite sequencing, ChIP and repli-seq, in order to assess the effect of reduced DNMT1 activity. While expectedly, global DNAme levels are decreased, they discovered a subset of PMDs gain DNA methylation, which they term hyperPMDs. There seems to be no impact on DNA replication timing, but the authors did go on to show that the de novo DNA methyltransferase, DNMT3A, is likely responsible for this counterintuitive increase in DNAme levels.

Reviewer #1 (Significance (Required)):

1. Overall, I found the data well-presented and diligently analyzed, as we have come to expect from the Sproul group. However, I am somewhat at a loss to understand both the rationale for the experimental set-up and the meaning of the results. The HCT116 cell line is already transformed but was treated as though it was a wild-type control. I was more curious to see how the PMD chromatin state and replication compare to a healthy cell.

We focused on the comparison between WT and DNMT1 KO cells as we wanted to understand the role of DNMT1 in maintaining the organisation of the cancer methylome.

To further understand the relevance of our findings in the context of carcinogenesis, we have now compared DNA methylation patterns in the normal colon to colorectal tumours.

We find that in colorectal tumours, HCT116 PMDs are significantly hypomethylated relative to HCT116 HMDs (*Rebuttal figure 1A*). This suggests that the domain-level organisation of HCT116 cells reflects that of clinical tumours. Furthermore, we find that DNA methylation variation between colorectal tumours is significantly higher in HCT116 hypermethylated PMDs than at other HCT116 H3K9me3-marked PMDs (*Rebuttal figure 1B*). This is consistent with methylation and chromatin organisation at hypermethylated PMDs being different from other PMDs in colorectal tumours *in vivo*.

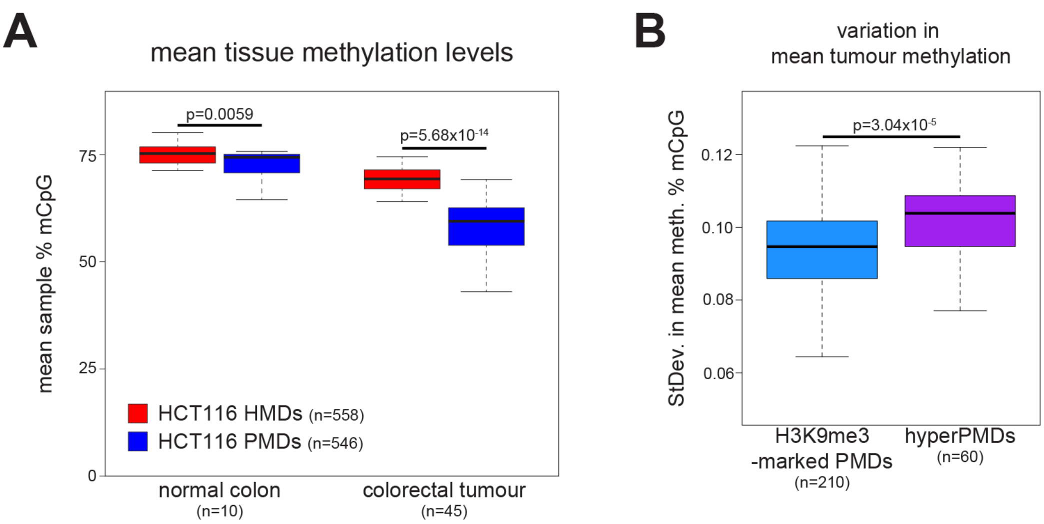

*Rebuttal figure 1. HCT116 PMDs are hypomethylated in colorectal tumours. (**A**) Boxplot of mean levels of PMD and HMD methylation for normal colon and colorectal tumour samples. (**B**) Boxplot of standard deviation in methylation level across colorectal tumours (n=45) for H3K9me3-marked PMDs and hypermethylated PMDs. For boxplots, lines = median; box = 25th–75th percentile; whiskers = 1.5 × interquartile range from box. All p-values are from two-sided Wilcoxon rank sum tests.*

We have included these analyses in a new *Figure 6* and section of the manuscript: '*Hypermethylated PMDs have variable methylation levels in colorectal tumours*'.

In addition, we have included discussion of the reviewer's point:

It is possible that DNMT localisation and function differ between normal and cancerous cells and thus our findings may not reflect the formation of PMDs during carcinogenesis. Indeed, differences between cancer cell line and tumour methylation patterns have previously been noted (67). However, here we also observe that PMDs in HCT116 cells reflect the domain-level methylome of colorectal tumours.

2. Moreover, the link between late replication and PMDs would indicate that a DNMT1 gain-of-function line would potentially be more interesting: could more increased DNMT activity rescue the PMDs, and how would this impact the chromatin and replication states? Perhaps this is not trivial to create; I do not know if simply overexpressing DNMT1 and/or UHRF1 could act as a gain-of-function.

We agree that a DNMT1 overexpression or a gain-of-function mutation cell line would be interesting to analyse and potentially informative as to the mechanism of PMD formation. However, as the reviewer notes, this is a complex experiment that could require the additional expression of partners such as UHRF1 or generation of an as-yet-unknown DNMT1 gain-of-function mutation. The full dissection of the implications of this separate experimental strategy would also entail the repetition of the majority of our experiments in DNMT1 KO cells and thus would be beyond the timescale of a manuscript revision.

In the revised manuscript, we have included the related experiment suggested by reviewer 2 (see *Reviewer 2*, *point 3*) and asked whether re-expression of DNMT1 in DNMT1 KO cells reverts DNA methylation patterns DNMT1 KO cells.

3. Nevertheless, the appearance of hyperPMDs was a curious finding worth publishing. However, it is unclear what the biological relevance is. There is no effect on replication timing, and no assessment on cell behavior (eg, proliferation assays). In other words, is DNMT3A performing some kind of

compensatory action, or is it just a curiosity? Below in the significance section, I have highlighted some additional specific points

The main significance of our findings that they support an alternative model for PMD formation during carcinogenesis. Given that PMD formation is thought to drive carcinogenesis through genomic instability (*Eden et al 2003, PMID: 12702868*) this is important to understand. The predominant hypothesis is that PMDs emerge in heterochromatin because their late replication timing leaves insufficient time for re-methylation following DNA replication (*Zhou et al 2018, PMID: 29610480* and *Petryk et al 2021, PMID: 33300031*). Our results, instead suggest that differential localization of *de novo* DNMTs is the critical factor in PMD formation as it protects euchromatin from hypomethylation during successive cell divisions.

Our results also suggest a potential role for DNMT3A in colorectal cancer, where it is less studied than DNMT3B.

Previous work on the role of DNMTs in colorectal cancer has largely focused on DNMT3B which is reported to be overexpressed in 15-20% of cases, often as a result of amplification (68-71). Its deletion or overexpression in mouse models also alter the rate of colorectal carcinogenesis (69, 72, 73) suggesting that it is a tumour promoting gene. DNMT3A expression in colorectal tumours is less studied but it is reported to be upregulated in at least some tumours (71, 74, 75). However, DNMT3A deletion inhibits colorectal carcinogenesis in a mouse model (75) suggesting it plays a functional role. Here, we provide evidence that it plays a key role in the organisation of the colorectal cancer methylome providing a basis for future studies as to its role in colorectal carcinogenesis using pre-clinical models that better reflect this process than cancer cell lines.

4. Why were DNMT3A and 3B transgenes used for ChIP instead of endogenous proteins? I know the authors cited work justifying this strategy, but this still merits explanation. Also, the expression level of transgenes compared to the endogenes was not shown (neither protein nor RNA level).

DNMT3A and B transgenes were used because antibodies against the endogenous proteins are not suitable for ChIP. Furthermore, performing these experiments using endogenously tagged proteins, would require the generation of 3 knock-in tagged lines (we have already generated HCT116 cells with tagged DNMT3B, *Masalmeh et al 2021, PMID: 33514701*).

As the reviewer suggests, we have added further explanation for our strategy to the results:

We then asked whether DNMT3A or DNMT3B localised differently in HCT116 and DNMT1 KO cells. DNMT3A and DNMT3B antibodies are unsuitable for ChIP. However, we have previously shown that ectopically expressed DNMT3B recapitulates the localisation of endogenous DNMT3B in HCT116 cells (31) suggesting that tagged, ectopically expressed enzymes can be used to understand DNMT3A and DNMT3B recruitment.

And discussed this potential caveat:

Here we have examined DNMT3A and DNMT3B localisation by performing ChIP-seq on ectopically expressed, tagged transgenes which we have previously shown are overexpressed relative to endogenous levels (32). However, we have also demonstrated that our overexpressed DNMT3B recapitulates the localization of the endogenously tagged protein to the genome (31) and our results reflect the consensus from previous studies (33, 34). Furthermore, ectopically expressed tagged-DNMT3A with HESJAS-causing PWWP mutations re-localises to polycomb-marked loci in cells (57). This mirrors gains in methylation at these loci in vivo in both HESJAS patients and mouse models (43,

65). The combined evidence therefore strongly suggests that ectopically expressed, tagged DNMT3A and DNMT3B can be used to understand the localisation of the endogenous proteins.

5. The DNMT3A binding profile appears as though it is on the edges of the PMDs and fairly depleted within (Fig 4A,D). Could the authors comment on this?

This is an interesting point. We note that although mean DNMT3A signal is indeed higher at the edges of hypermethylated PMDs than inside these domains, its levels within the central part of these PMDs are both above background and that observed in HCT116 cells. As suggested by reviewer 3, this could be consistent with H3K36me2 and DNMT3A spreading in from the boundaries of hypermethylated PMDs in DNMT1 KO cells and we discuss this possibility further below (see *reviewer 3*, *point 1*).

6. A more compelling experiment would be to assess the loss of DNMT3A genetically. How would this affect PMD DNA methylation? Maybe in this case there would be an effect on replication timing. Could a KO or KD (eg, siRNA) strategy be employed to assess this on top of either the HCT116 or DNMT1 KO.

We agree with the reviewer that this experiment would further define the role of DNMT3A at PMDs. However, the revision period for this study was insufficient for us to generate this data. We have added a point to the discussion noting the utility of this experiment:

Future studies using time-resolved models and manipulation of DNMT3A will be required to understand the interplay between DNMT1 loss, changes in chromatin structure and de novo DNMT localisation.

7. What is the major H3K36me2 methylatransferase in these cells? Could an Nsd1 KO or KD strategy be used, for example, to show that indeed H3K36 methylation is required for HyperPMDs? This would complement the DNMT3A experiment above.

We have now analysed the expression levels of H3K36 methyltransferases in HCT116 and DNMT1 KO cells. We find evidence that all 5 well-known H3K36 methyltransferases are expressed in HCT116 cells but that none are differentially expressed in DNMT1 KO cells (*Rebuttal figure 2*). It is therefore unclear which enzyme(s) might be responsible for H3K36me2 deposition at hypermethylated PMDs. As delineation of this enzyme would require multiple perturbation and sequencing experiments, it is beyond the timescale of a manuscript revision.

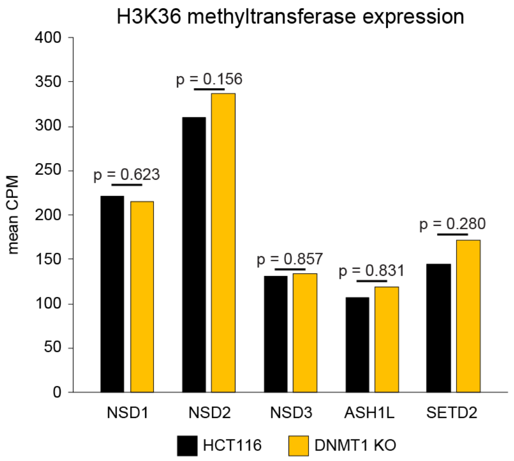

Rebuttal figure 2. HCT116 cells express multiple H3K36 methyltransferases. Barplot of mean expression levels for putative mammalian H3K36 methyltransferases in HCT116 and DNMT1 KO cells.

Expression levels are counts per million (CPM) derived from RNA-seq. Mean expression levels are derived from 9 and 4 independent cultures of HCT116 and DNMT1 KO cells respectively. P-values are from F-Tests adjusted for multiple testing using the Benjami-Hochberg approach.

These data are included *Figure S7D* and discussed in the manuscript:

To understand whether increases in the levels of an H3K36me2 methyltransferase might be responsible for increases in this mark at hypermethylated PMDs in DNMT1 KO cells, we examined RNA levels of putative H3K36 methyltransferases in our RNA-seq data. Robust evidence exists for 5 different human H3K36 methyltransferases: NSD1, NSD2, NSD3, ASH1L, SETD2 (39), and their deletion in human cells entirely removes H3K36 methylation (40). All 5 were expressed in both HCT116 and DNMT1 KO cells but their RNA levels were not significantly different between the two cell lines (Figure S7D) meaning that it is unclear which might be responsible for deposition of H3K36me2 at hypermethylated PMDs.

8. Based on Figure 2C, it seems that a general predictive pattern of hyperPMDs is H3K9me3-enriched and H3K27me3-depleted. Is this an accurate interpretation? Given the authors' expertise in the relationship between DNMT3A and polycomb, could they perhaps give an explanation for this phenomenon?

The reviewer is correct. In HCT116 cells, those PMDs that become hypermethylated in DNMT1 KO cells are marked by H3K9me3 and are H3K27me3-depleted (except at their boundaries). DNMT3A can be recruited to polycomb-marked regions associated with H3K27me3 through interaction of its N-terminal region with H2AK119ub. However, this mark is depleted from hypermethylated-PMDs in DNMT1 KO cells (see *Figure S6D*) meaning that this recruitment pathways is unlikely to explain DNMT3A's localisation to hypermethylated PMDs in DNMT1 KO cells. We have included discussion of this possibility in the revised manuscript:

We and others have reported that DNMT3A is also recruited to the polycomb-associated H2AK119ub mark through its N-terminal region (53-57). However, we do not observe the polycomb-associated H3K27me3 mark, which is generally tightly correlated with H2AK119ub (58), at hypermethylated PMDs. Furthermore, DNMT3A's localisation is predominantly driven by PWWP-dependent recruitment to H3K36me2 unless its PWWP domain is mutated (57), suggesting that H2AK119ub does not play a role in the recruitment of DNMT3A to hypermethylated PMDs.

9. This is a minor point, but calling the DNMT1 mutant a "KO" seemed a bit misleading, as it is a truncation mutant. Perhaps there is a more accurate way to describe this line.

We have added additional text to clarify the nature of these cells in the revised manuscript and noting that we refer to these cells as DNMT1 KO cells for the sake of brevity in the manuscript and figures:

As complete removal of DNMT1 in somatic cells is lethal, these cells express a truncated DNMT1 lacking exons corresponding to the DMAP and PCNA-interacting domains, to around 20% of the level of DNMT1 in HCT116 cells (27, 28). They are therefore strictly a truncated, hypomorphic line, but for the sake of brevity we refer to these cells as DNMT1 KO cells throughout the manuscript.

Reviewer #2 (Evidence, reproducibility and clarity (Required)):

In this study, Kafetzopoulos et al. investigated the role of DNMT1-mediated methylation maintenance in cancer partially methylated domains (PMDs) using DNMT1 knockout HCT116 colorectal cancer cells. They used a range of sequencing-based approaches, including whole-genome bisulfite sequencing (WGBS), chromatin immunoprecipitation sequencing (ChIP-seq), and replication timing

sequencing (Repli-seq), to define the dynamics of DNA methylation loss and gain in PMDs during DNA synthesis. Interestingly, they demonstrate that specific PMDs marked by H3K9me3 undergo a gain of DNA methylation in DNMT1-deficient HCT116 cells. This increase in methylation is associated with the loss of H3K9me3, an enrichment of H3K36me2, and the recruitment of DNMT3A. These findings suggest that de novo methyltransferase activity plays a critical role in determining which genomic regions become PMDs in cancer.

The authors use a comprehensive and well-controlled set of sequencing-based techniques. While the sequencing depth for DNA methylation is somewhat limited, the inclusion of multiple biological replicates strengthens the reliability of the data. The study effectively integrates multiple layers of epigenomic information, providing a nuanced view of PMD regulation in the context of DNMT1 loss.

Overall, this paper provides valuable insights into the epigenetic regulation of PMDs in cancer, and its conclusions are well supported by the data. It significantly advances our understanding of how DNMT1 loss reshapes the epigenome and highlights the interplay between de novo and maintenance methylation mechanisms in cancers.

Reviewer #2 (Significance (Required)):

General assessment

-The main strength of the study lies in the clear presentation of the data, which follows a cohesive and well-defined storyline.

-The authors demonstrate that both hypomethylated and hypermethylated domains occur at the late replication stage. They further investigate the dynamics of histone modifications and DNA methylation, focusing on the acquisition and loss of these marks, particularly in relation to DNMT3A and DNMT3B.

Limitation

1. Although the study is compelling, its primary limitation is the correlative nature of most of the data. While the high-level representations (e.g., tracks, heat maps) are convincing, the study would have been more informative if it had explored the impact of these changes on a specific set of genes or regions critical to cancer initiation and progression. For example, in the DNMT1 knockout model, how does the loss of H3K9me3, the gain of H3K36me2, and the recruitment of DNMT3A in hypermethylated PMDs affect the expression of key genes involved in colorectal cancer?

To understand how the remodelling of DNA methylation and chromatin structure in DNMT1 KO cells affects gene expression, we have now compared gene expression in DNMT1 KO cells to HCT116 cells. In brief, we find that in DNMT1 KO cells, genes in PMDs are more likely to be upregulated relative to those in HMDs (*Rebuttal figure 3A*) and that genes in hypermethylated PMDs are upregulated to a significantly greater level than those in other H3K9me3-marked PMDs (*Rebuttal figure 3B*). This suggests that the changes we report at hypermethylated PMDs can impact on gene expression.

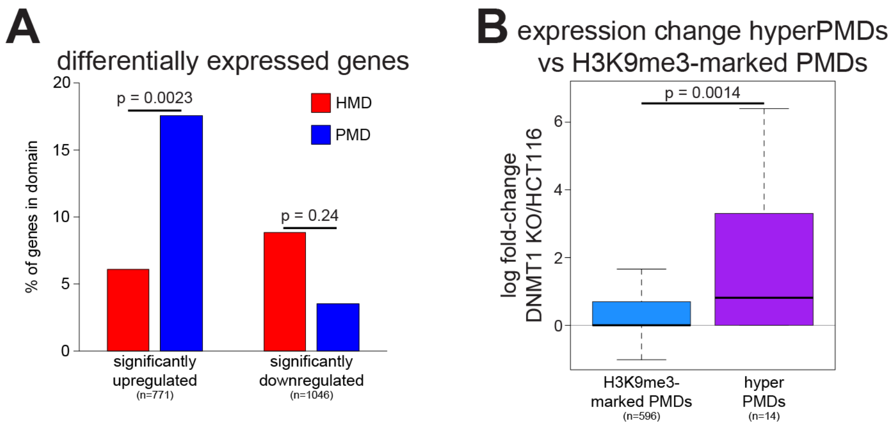

*Rebuttal figure 3. PMD-associated genes are upregulated in DNMT1 KO cells. (**A**) Barplot showing the % of significantly differentially expressed genes in PMDs and HMDs (n = 771 upregulated and 1,046 downregulated genes). (**B**) Boxplot showing log fold changes for DNMT1 KO vs HCT116 cells for genes located in hypermethylated PMDs versus other H3K9me3-marked PMDs. In all plots, expression values were calculated form n = 9 and 4 independent cultures of HCT116 and DNMT1 KO cells respectively. In **D**, n = 596 genes for H3K9me3-marked PMDs and 14 genes for hypermethylated PMDs. P-values shown are from two-sided Fisher's exact tests (**B**) and Wilcoxon rank sum tests (**D**). For the boxplot, lines = median; box = 25th–75th percentile; whiskers = 1.5 × interquartile range from box.*

This analysis detailed in full in a new *Figure S9* and discussed more fully in a new section of the revised manuscript '*Genes in hypermethylated PMDs are upregulated*'.

Given the small number of genes (n = 14 within hypermethylated PMDs), we cannot make any robust statements regarding the potential role of remodelling of DNA methylation at PMDs in carcinogenesis. Delineation of the impact of these changes in gene expression will require future work in pre-clinical models of colorectal carcinogenesis.

Additional experiments that could provide deeper insights

2. Cross-validation in other cancer cell lines would have enable to define if these signatures are observed beyond HCT116.

As detailed above in response to reviewer 1 (*point 1*), the revised manuscript includes a new section describing analyses from clinical colorectal tumour samples (*Hypermethylated PMDs have variable methylation levels in colorectal tumours*). This demonstrates that hypermethylated PMDs behave differently to other PMDs in colorectal tumours suggesting that they are not just a feature of HCT116 cells and their DNMT1 KO derivatives.

3. Are the observed signatures permanent, or could they be reversed by reinstating the full activity of DNMT1? Since DNMT1 might be dysregulated but never completely deleted.

As the reviewer suggests, we have analysed the impact of expressing DNMT1 in DNMT1 KO cells in the revised manuscript.

In brief, we observe that re-expression of DNMT1 leads to increases in overall mean DNA methylation levels by 4.83% compared to DNMT1 KO cells. However, levels are not restored to that of HCT116 cells (*Rebuttal figure 4A*) and we do not observe reversion of methylation levels at hypermethylated PMDs (*Rebuttal figure 4A-B*).

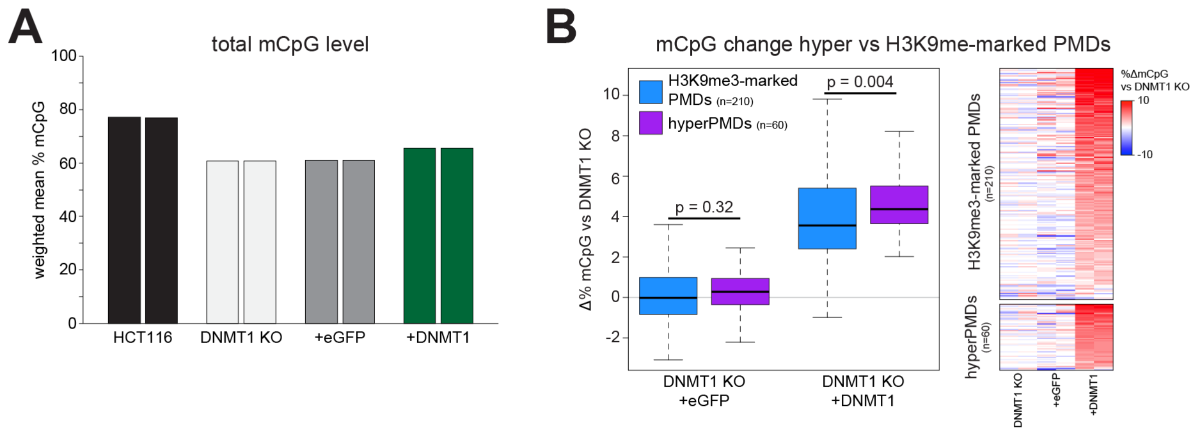

*Rebuttal Figure 4. DNMT1 rescue leads to gains of methylation throughout the genome. (**A**) Barplot of total CpG DNA methylation levels estimated by Nanopore sequencing from all aligned reads. For each condition, two independent replicate cultures are shown. (**B**) Boxplots (left) and heatmaps (right) showing differential DNA methylation between DNMT1 KO and DNMT1 KO cells expressing either eGFP or DNMT1 at hypermethylated PMDs and other H3K9me3-marked PMDs. Boxplot shows the mean values of two independent replicate cultures. Barplot and heatmaps show replicates separately. n = 210 domains for H3K9me3-marked PMDs and 60 for hypermethylated PMDs. In all plots: +eGFP = DNMT1 KO expressing eGFP and +DNMT1 = DNMT1 KO expressing DNMT1. For the boxplot, lines = median; box = 25th–75th percentile; whiskers = 1.5 × interquartile range from box. P-values are from two-sided Wilcoxon rank sum tests.*

These data are described in more detail as a new *Figure S8* and in the manuscript:

To understand whether this remodelling of chromatin structure was permanent, we expressed human DNMT1 in DNMT1 KO cells and assayed genome-wide DNA methylation levels using Nanopore sequencing. DNMT1 expression led to a mean 4.83% increase in DNA methylation levels that was not observed in control cells expressing eGFP (where the mean increase was 0.22%, Figure S8A). However, methylation levels did not return to those of HCT116 cells (Figure S8A) consistent with the predominant role of DNMT1 being a maintenance rather than a de novo methyltransferase (36). These gains of methylation were observed throughout the genome with a bias towards slightly greater increases in HMDs versus PMDs (Figure S8B-C, p = 1.85x10-5, Wilcoxon rank sum test). However, we did not observe a reversion of methylation levels at hypermethylated PMDs which gained slightly more methylation than other H3K9me3-marked PMDs (Figure S8D, p = 0.04, Wilcoxon rank sum test). This suggests that the generation of DNMT1 KO cells led to a reconfiguration of the chromatin landscape at hypermethylated PMDs that does not revert upon reintroduction of DNMT1. Furthermore, as Nanopore sequencing can distinguish methylcytosine from hydroxymethylcytosine (41), unlike WGBS (42), these data confirm that the changes we report are due to differences in the abundance of methylcytosine.

And in the discussion:

Taken together, our observations suggest that hypermethylated PMDs result from a reconfiguration of chromatin state in these regions during the generation of DNMT1 KO cells…. In support of this hypothesis, we find that re-expressing of full-length DNMT1 does not lead to a reversion of methylation levels at hypermethylated PMDs.

4. Use knockdown and overexpression experiments to track the dynamics and occurrence of these molecular events over time, providing insight into the progression and reversibility of epigenetic changes.

As the reviewer suggests, we have now analysed the behaviour of domain-level DNA methylation in HCT116 cells in an AID degron-tagged DNMT1 line.

Briefly, upon DNMT1-removal, we observe genome-wide loss of methylation (*Rebuttal Figure 5A*). Analysis of the kinetics of this loss at different genomic domains shows that DNA methylation is lost more rapidly from PMDs than HMDs (*Rebuttal Figure 5B*). This is consistent with our model that greater *de novo* DNMT activity at HMDs compared to PMDs. Furthermore, this sensitive analysis reveals that hypermethylated PMDs lose methylation more slowly than other H3K9me3-marked PMDs (*Rebuttal Figure 5C*) suggesting that they could be associated with higher *de novo* DNMT activity even in HCT116 cells.

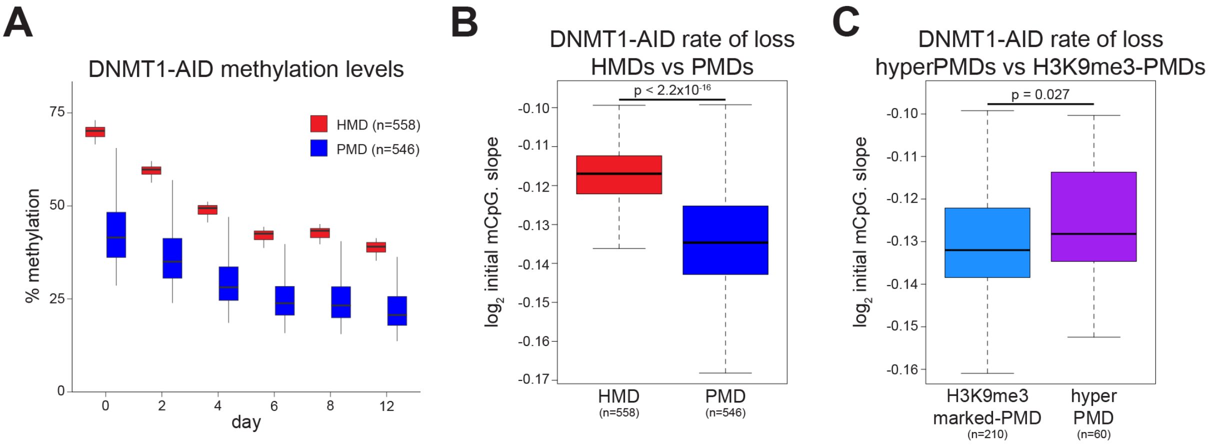

*Rebuttal figure 5. Hypermethylated PMDs lose DNA methylation more slowly following DNMT1 removal. (**A**) Boxplot of mean methylation level for PMDs and HMDs following degradation of DNMT1 in AID-DNMT1 cells. (**B**, **C**) Boxplot of rate of loss of DNA methylation in AID-DNMT1 cells for PMDs and HMDs (**B**) and hypermethylated PMD compared to other H3K9me3-marked PMDs (**C**). In plots, n = 558 domains for HMDs, 546 domains for PMDs, 210 domains for H3K9me3-marked PMDs and 60 for hypermethylated PMDs. For boxplots, lines = median; box = 25th–75th percentile; whiskers = 1.5 × interquartile range from box. All p-values are from two-sided Wilcoxon rank sum tests.*

These new data are fully detailed in new *Figure S5* and in the manuscript:

To further understand how de novo DNMT activity is distributed in the cancer genome, we analysed DNA methylation levels following acute DNMT1 removal from a DNMT1 degron HCT116 line, AID-DNMT1 cells (35). DNMT1 removal leads to genome-wide hypomethylation (35). In the absence of de novo activity this loss would be expected to follow exponential decay dynamics irrespective of initial level (Figure S5A). However, differential de novo DNMT activity between genomic domains would alter their relative kinetics (Figure S5A). Specifically, higher levels of de novo DNMT activity i) slows DNA methylation loss and ii) results in DNA methylation levels plateauing at a higher level (Figure S5A).

We analysed a time-course of WGBS following DNMT1 removal from AID-DNMT1 cells, observing that both HMDs and PMDs lost methylation and that levels plateaued after 6 days. However, HMDs lost methylation at a significantly slower rate than PMDs and remained at a significantly higher DNA methylation level following completion of the time-course (Figure S5B-C, both p < 2.2x10-16, Wilcoxon rank sum tests) suggesting higher de novo DNMT activity in HMDs than PMDs. To understand whether de novo DNMT activity in HCT116 cells differed at hypermethylated PMDs relative to other PMDs, we also analysed their kinetics. Relative to other H3K9me3-marked PMDs, the observed rate of DNA methylation loss was significantly slower at hypermethylated PMDs and they retained significantly higher level of DNA methylation (Figure S5D, p = 0.027 and 2.77x10-4

respectively, Wilcoxon rank sum tests). This is consistent with hypermethylated PMDs having higher de novo DNMT activity than other PMDs in HCT116 cells.

Advances

-The study provides new insights into the establishment of PMD types in colorectal cancer cell lines.

-These findings contribute both conceptually and mechanistically to our understanding of how DNA methylation patterns are regulated during cancer development.

Audience:

-This study will appeal to a broad audience, from researchers primarily focused on epigenetics and cancer biology to those interested in the mechanistic underpinnings of DNA methylation and its role in cancer progression. It will also be relevant to those exploring therapeutic strategies targeting epigenetic regulators in cancer.

We thank the reviewer for their kind comments on our manuscript.

Reviewer #3 (Evidence, reproducibility and clarity (Required)):

Summary: Cancer is linked to the acquisition of an atypical DNA methylation landscape, with broad domains of partial DNA methylation (termed PMDs). This study investigates PMDs in a colorectal cancer cell line and evaluates the contribution of DNMT1 in maintaining PMDs, using a DNMT1 KO line. The authors find that PMDs preferentially lose DNA methylation upon loss of DNMT1, but they find a number of domains that paradoxically gain DNA methylation (hyperPMDs). They attribute this gain of methylation to the action of DNMT3A through the accumulation of H3K36me2 and loss of heterochromatin mark H3K9me3. Together this work sheds light on the dynamic mechanisms regulating the atypical DNA methylation landscape in colorectal cancer cells.

1. General comments: The introduction is informative and well written. Additionally, the work is rigorously done and analyses are clear. However, the conclusions and summary figure largely focus on the relationship between PMDs with H3K9me3 and H3K36me2, but I think the role for H3K27me3 should be revisited based on the results presented. H3K9me3 is present at PMDs and hyperPMDs, but H3K27me3 level appears to be a much more defining feature of whether they lose or gain methylation upon loss of DNMT1 (Figure 2, Figure S2C- D). There is a reported interplay between PRC2 and DNMT3A activity at DNA methylation valleys in other cell contexts (e.g., mouse embryogenesis, hematopoietic cells), so couldn't H3K27me3 be performing a 'boundary' function at PMDs and when sufficiently low, permits spread of H3K36me2 in the absence of DNMT1? I think it is worth further exploring the H3K27me3 data.

As the reviewer suggests we have conducted further analysis of the relationship between H3K27me3 and H3K36me2. This demonstrates that H3K27me3 and H3K36me2 are negatively correlated in the genome in both HCT116 and DNMT1 KO cells (*Rebuttal figure 6*) aligning with multiple studies showing that H3K36me2 restricts H3K27me3 deposition in the genome (*Streubel et al 2018, PMID: 29606589, Shirane et al 2020, PMID: 32929285 and Farhangdoost et al 2021, PMID: 3362635*).

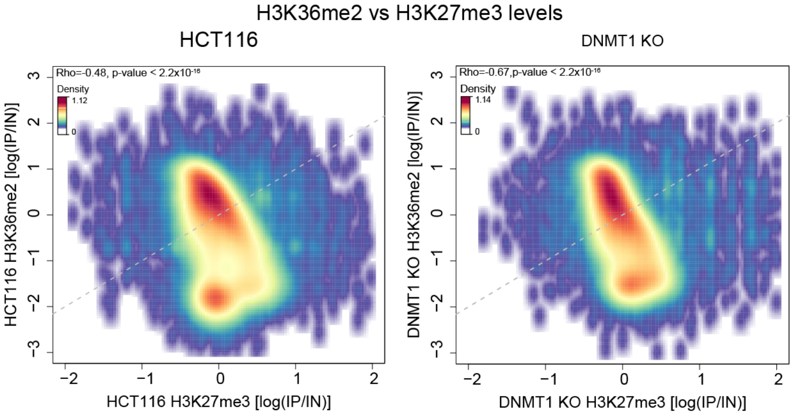

Rebuttal Figure 6. H3K36me2 is anticorrelated with H3K27me3. Density scatters plot showing genome-wide correlation between H3K36me2 and H3K27me3 levels in HCT116 (left) and DNMT1 KO cells (right). ChIP-seq data are normalised log IP/IN in 10kb windows from the mean of n=2 replicates. Spearman's correlation (Rho) and associated p-value is shown.

These data are included in a new *Figure S7B* in the manuscript.

As also suggested, we have analysed the presence of H3K27me3 at the boundaries of PMDs. We find that H3K27me3 is indeed present at the boundaries of both H3K9me3-marked and hypermethylated PMDs in HCT116 cells with levels being slightly lower at hypermethylated PMDs (*Rebuttal Figure 7*). In DNMT1 KO cells, we see that boundary H3K27me3 levels decrease to background but remain above background at other H3K9me3-marked PMDs (*Rebuttal Figure 7*).

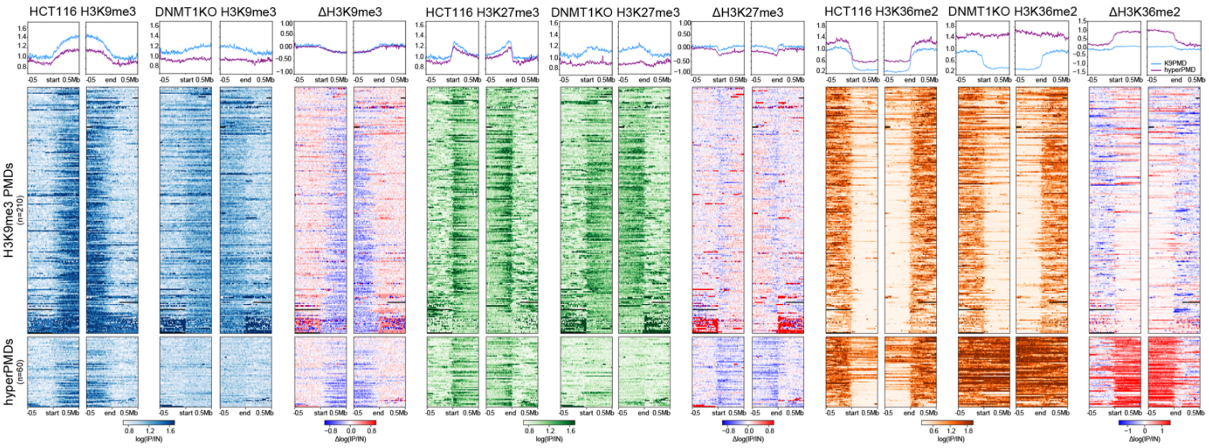

Rebuttal Figure 7. Analysis of histone modifications at PMD boundaries. Heatmaps and pileup plots of HCT116 and DNMT1 KO histone modification levels for hypermethylated PMDs (n= 60 domains) and all other K9-marked PMDs (n= 210 domains). Shown are levels aligned to the start and end of each domain relative to the genome plus strand -/+ 0.5Mb. ChIP-seq data are mean log- normalised IP/IN. Domains are ranked based on their mean level of DNA methylation in HCT116 cells. All histone ChIP-seq data shown are derived from the mean of two biological replicates

As the reviewer suggests, this may allow spreading of H3K36me2 into hypermethylated PMDs. Robust testing of this hypothesis will, however, require future work in time-resolved systems. We have added a statement to the discussion to explain this:

Future studies using time-resolved models and manipulation of DNMT3A will be required to understand the interplay between DNMT1 loss, changes in chromatin structure and de novo DNMT localisation.

2. Additionally, a key point that is illustrated in the summary figure, is the localization of H3K36me2 at HMDs and its mutual exclusivity with H3K9me3 (a mark typically associated with high DNA methylation). However, because the H3K36me2 is introduced quite late in the analysis, I feel that a rigorous evaluation of its enrichment and anti-correlation with H3K9me3 at highly methylated domains (HMDs) is missing.

As the reviewer suggests, we have examined the global relationship between H3K9me3 and H3K36me2, reinforcing that they are mutually exclusive in the genomes of both HCT116 and DNMT1 KO cells (*Rebuttal figure 8*).

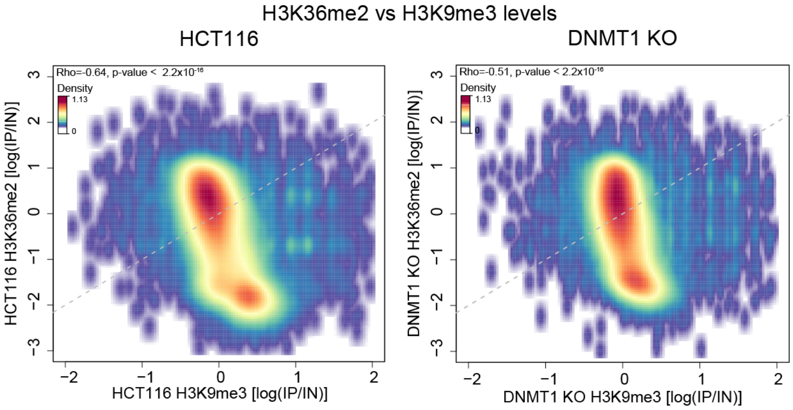

Rebuttal Figure 8. H3K36me2 is anticorrelated with H3K9me3. Density scatters plot showing genome-wide correlation between H3K36me2 and H3K9me3 levels in HCT116 (left) and DNMT1 KO cells (right). ChIP-seq data are normalised log IP/IN in 10kb windows. Spearman's correlation (Rho) and associated p-value is shown.

These are included in a new *Figure S7C* and discussed in the manuscript:

They also suggest that DNMT3A re-localisation occurs following disruption of heterochromatin caused by DNMT1 loss. Indeed losses of H3K9me2/3 are previously reported in DNMT1 KO cells (59) and acute depletion or inhibition of DNMT1 reduces H3K9me3 levels and alters heterochromatin compartmentalisation (60, 61). This could allow H3K36 methyltransferases access and deposition of H3K36me2. Further evidence of interplay between these two marks is provided by another recent study demonstrating that perturbation of H3K36me2 leads to alterations in H3K9me3 distribution (40).

3. In general, I also found that I was jumping between figures a lot and needed to look at the supplements to gain the full picture. It may be beneficial to re-organize the figures.

During the process of revising the manuscript it has been reorganised in accordance with reviewer comments and we hope that it is now clearer.

Specific Comments/Questions:

4. An expanded explanation of the truncated DNMT1 in the DNMT1 KO cells would be helpful for context

As suggested by the reviewer, we have included an expanded introduction to these cells in the revised manuscript:

To understand the role of DNMT1 in shaping the cancer epigenome at the domain level, we compared the methylomes of HCT116 cells to a published DNMT1 knock-out derivative (26) using Whole Genome Bisulfite Sequencing (WGBS, HCT116 mean coverage= 1.6x, DNMT1 KO mean coverage= 2.4x). As complete removal of DNMT1 in somatic cells is lethal, these cells express a truncated DNMT1 lacking exons corresponding to the DMAP and PCNA-interacting domains, to around 20% of the level of DNMT1 in HCT116 cells (27, 28). They are therefore strictly a truncated, hypomorphic line, but for the sake of brevity we refer to these cells as DNMT1 KO cells throughout the manuscript.

5. Does the DNMT expression in HCT116 cells reflect the levels seen in primary colorectal cancers? Hence, do you think these cultured cells reflect aspects of DNA methylation dynamics that would be seen in tumors?

As discussed in response to reviewer 1 (*point 1,* above), we have now examined the methylation status of HCT116 PMDs in colorectal tumours showing that our findings in HCT116 are reflected the organisation of the methylome in clinical colorectal tumours. These data are included in a new manuscript section '*Hypermethylated PMDs have variable methylation levels in colorectal tumours*' and *Figure 6*.

We have also added discussion detailing current knowledge regarding the expression level and role of DNMT3A and DNMT3B in colorectal cancer:

Previous work on the role of DNMTs in colorectal cancer has largely focused on DNMT3B which is reported to be overexpressed in 15-20% of cases, often as a result of amplification (68-71). Its deletion or overexpression in mouse models also alter the rate of colorectal carcinogenesis (69, 72, 73) suggesting that it is a tumour promoting gene. DNMT3A expression in colorectal tumours is less studied but it is reported to be upregulated in at least some tumours (71, 74, 75). However, DNMT3A deletion inhibits colorectal carcinogenesis in a mouse model (75) suggesting it plays a functional role. Here, we provide evidence that it plays a key role in the organisation of the colorectal cancer methylome providing a basis for future studies as to its role in colorectal carcinogenesis using pre-clinical models that better reflect this process than cancer cell lines.

6. Although DNMT3A/B mRNA levels are similar between DNMT1 KO and HCT116 cells, is the protein abundance altered? I think there would be value in showing a Western blot analysis, as the loss of DNMT1 protein may alter the stability of the de novo DNMTs. Is a similar level of expression of the ectopic T7-DNMT3A and T7-DNMT3B achieved in HCT116 and DNMT1 KO cells? A western blot showing this would also be valuable.

We have now examined DNMT3A and DNMT3B levels by Western blots. This confirms that DNMT3A protein levels are similar between HCT116 and DNMT1 KO cells (*Rebuttal Figure 9A)*. In addition, we observe decreases in the level of the catalytically active DNMT3B isoform, DNMT3B2, and similar levels of the catalytically inactive accessory isoform DNMT3B3 (*Rebuttal Figure 9B*).

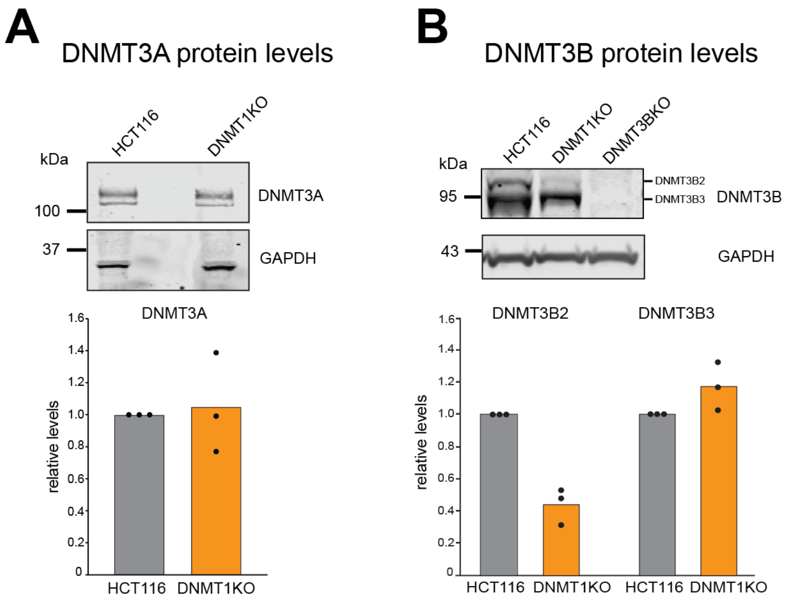

Rebuttal figure 9. Analysis of DNMT3A and DNMT3B protein levels. A. DNMT3A protein levels are similar in HCT116 and DNMT1 KO cells. B. DNMT3B isoform levels are altered in DNMT1 KO cells. In both, Western blots are a representative example. Bar heights indicate mean levels in protein extracts from 3 independent cell cultures normalised to HCT116 cells. Individual points indicate the level of each replicate. DNMT3BKO = DNMT3B knockout HCT116 cells to confirm antibody specificity.

We have included these data as a new *Figure S4-C* and added discussion of the results to the revised manuscript:

However, only small differences in the RNA levels of both DNMT3A and DNMT3B were observed in DNMT1 KO cells relative to HCT116 cells (Figure S4A). We then asked whether this was reflected at the protein level using Western blots, finding that DNMT3A was expressed to similar levels in HCT116 and DNMT1 KO cells (Figure S4B). In contrast, catalytically active DNMT3B2 was downregulated in DNMT1 KO cells but its catalytically inactive adaptor isoform, DNMT3B3, remained similarly expressed (Figure S4C). This suggests DNMT3A levels are not greatly altered in DNMT1 KO cells, but that DNMT3B2 is downregulated.

As the reviewer also requests, we provide Western blots showing that ectopically expressed DNMT3A and DNMT3B are similarly expressed in HCT116 and DNMT1 KO cells (*Rebuttal Figure 10*).

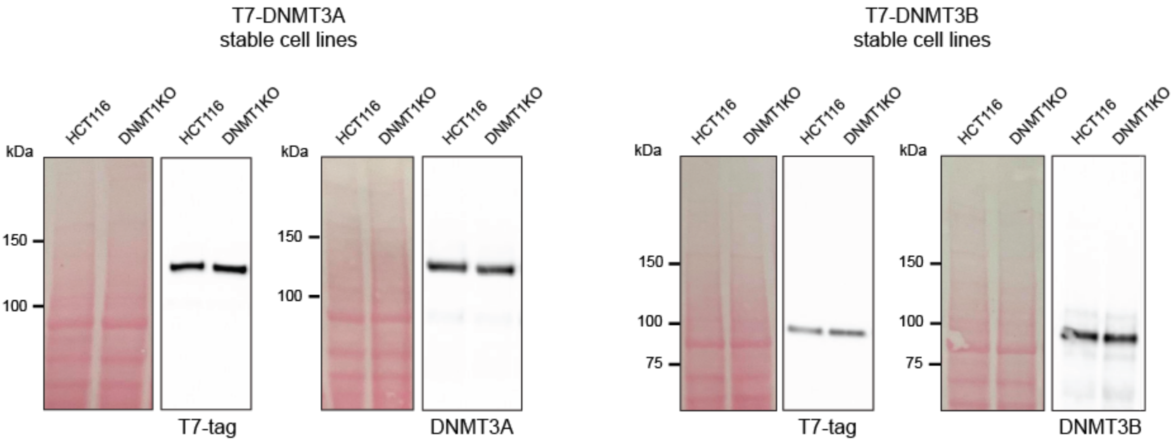

Rebuttal figure 10. Ectopic DNMT3A and DNMT3B are similarly expressed in HCT116 and DNMT1 KO cells. Western blots from cell lines expressing ectopic DNMT3A1 and DNMT3B2 used for T7-ChIP-seq. Antibodies used are indicated below blots. Photographs of the gel Ponceau staining are shown to indicate equivalent loading.

7. Do you think that the increase in DNMT3A over HyperPMD compared to H3K9me3-marked PMDs is related to an increase in protein bound at these domains or an altered residence time?

The reviewer makes an interesting point. We have added discussion of this possibility to the revised manuscript:

Given that ChIP-seq signal is affected by residence time (66), it is possible that our findings of increased DNMT3A ChIP-seq signal in hypermethylated PMDs could reflect altered residence time rather than increased DNMT3A localisation.

8. It would also be valuable to move the plot showing levels of DNMT3A/3B at HMDs, from the S4C/D to the main Figure 4, for reference. It would also be interesting to see the enrichment of DNMT3A/B at all PMDs (not just H3K9me3-marked PMDs).

We have modified the manuscript in accordance with this suggestion. HMDs are now included in *Figures 4B-C* and the enrichment of DNMT3A and DNMT3B at all PMDs is shown in *Figures S4E-F*.

9. It appears that the same genomic locus is used multiple times across figures Fig 1A, Fig 2B, Fig 3A, Fig 4A, Fig 5B to illustrate the trends reported from the global analyses. While this has value in showing the dynamics across datasets at this region, I think it is important to illustrate that these trends can be observed elsewhere. Please add or replace some plots with additional loci. Furthermore, please add the genomic region coordinates to the figure or figure legend.

We have shown a single locus for consistency and to not overcomplicate figures which already contain multiple panels. For the reviewer we include additional loci below showing that we observe similar patterns to the locus we show in the manuscript (*Rebuttal Figure 11*).

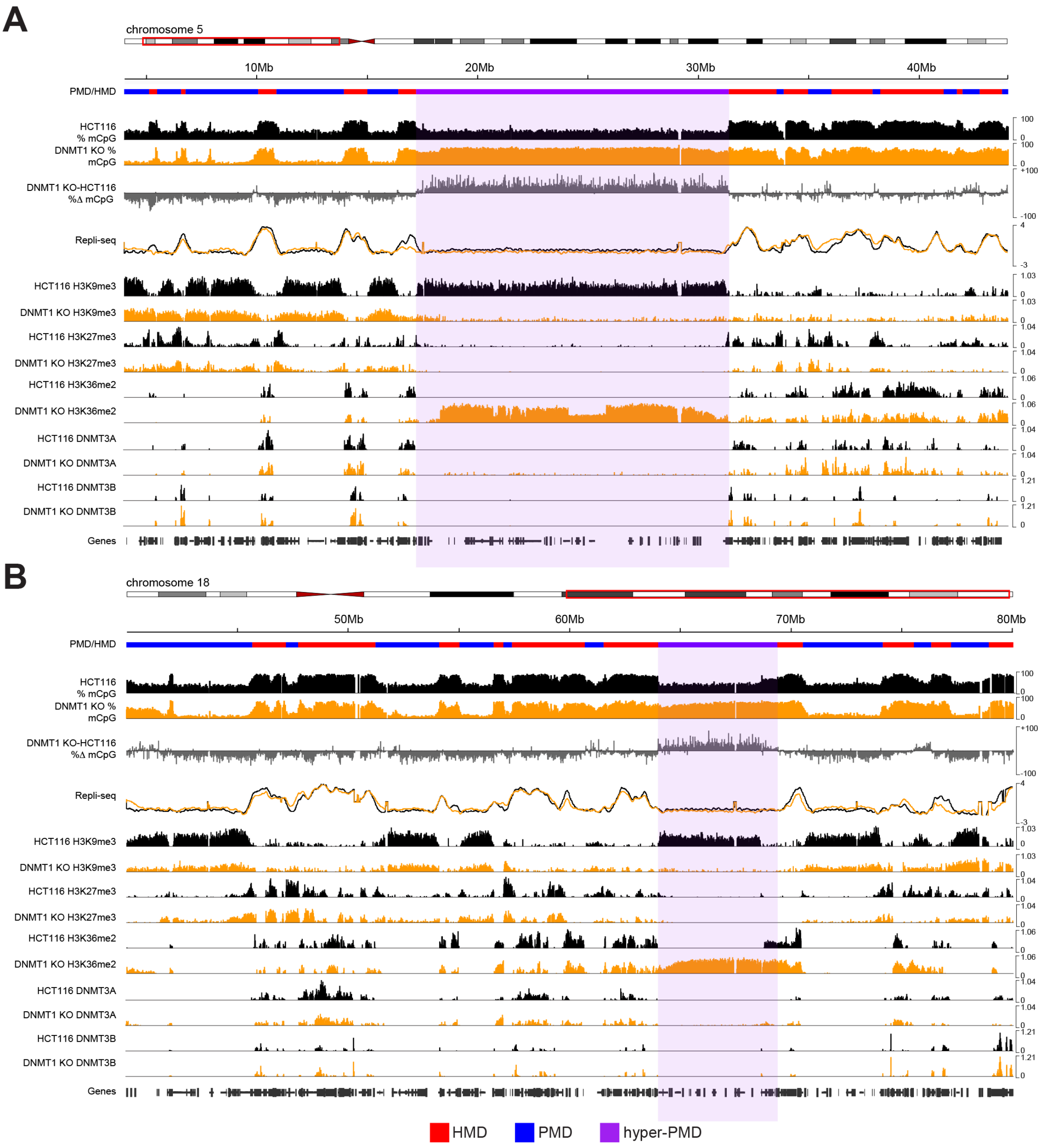

*Rebuttal Figure 11. Genome browser shots of additional chromosomal loci showing replication of key findings on chromosome 5p (**A**) and chromosome 18q (**B**). DNA methylation levels are plotted in 10 kb genomic windows. ChIP-seq tracks are normalised log10 IP/IN. Replication timing data are loess smoothed repli-seq early/late ratios over 10 kb. All histone ChIP-seq and repli-seq data shown are derived from the mean of two biological replicates. Boxes highlight hypermethylated PMDs. Chromosomal co-ordinates shown are from hg38: (**A**) chr5:4,000,000-44,000,000 and (**B**) chr18:40,000,000-80,000,000.*

In the revised manuscript, we have also ensured that the precise co-ordinates stated in the figure legends.

10. The ChIP-seq data is quantified as IP/input. This quantitation can be prone to introducing artefacts into analyses if the input coverage is substantially uneven over AT-rich regions or CpG islands, or if the sequencing depth is insufficient. I would encourage the authors to check that the

trends observed are still present if quantified without correcting against the inputs. If using IP/input, in the supplementary figures, I think it would be valuable to show the uncorrected quantitation of inputs across PMDs, to demonstrate that there is even coverage and this isn't contributing to any of the changes observed.

To demonstrate that variation in ChIp-seq input signal is unlikely to explain our results, particularly in the case of the case of DNMT3A where enrichments across hypermethylated PMDs are relatively low level, we provide additional genome browser plots:

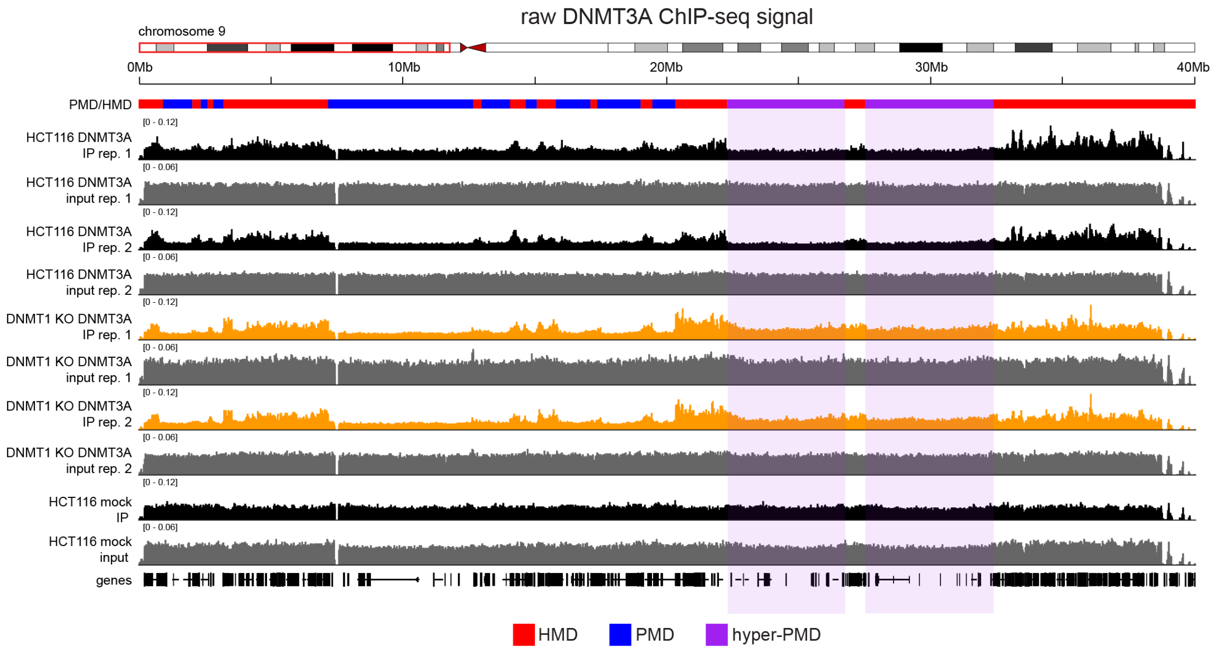

Rebuttal Figure 12: Representative chromosomal locus showing raw DNMT3A ChIP-seq signal. ChIP-seq is shown as separate tracks for input and IP from HCT116 and DNMT1 KO cells. Tracks are log read counts. Boxes highlight hypermethylated PMDs. Chromosomal co-ordinates shown are hg38 chr9:1-40000000.

11. Generally, the n numbers for different groups of probes can be confusing and increased clarity would be helpful.

In the revised manuscript, we have ensured that the nature of the n numbers given is clarified in the figure legends.

Reviewer #3 (Significance (Required)):

This study adds to the accumulating body of evidence that DNMT3A recruitment is mediated primarily through H3K36me2 across cell contexts, shedding light on the interplay between histone modifications and de novo DNA methylation. Understanding these mechanisms is important to appreciate the role for DNMT3A in establishing DNA methylation in development and disease contexts. It does remain unclear why, upon loss of DNMT1 in colorectal cancer cells, some PMDs accumulate H3K36me2 and consequently DNA methylation, while others do not. Further study into the chromatin dynamics will be valuable in understanding determinants of the DNA methylation landscape in cancer.

We thank the reviewer for their insightful comments and believe that our proposed revisions will further clarify the points they raise.
